# Supplementary material for: Design and Development of Lysyl tRNA Synthetase Inhibitors, for the Treatment of Tuberculosis
Source: J Med Chem. 2025 Aug 1;68(15):16459–82. doi: 10.1021/acs.jmedchem.5c01331 (PMC12362622; doi:10.1021/acs.jmedchem.5c01331)
Supplement: Supplementary file 1 [file jm5c01331_si_001.pdf]

Supporting Information

Design and development of lysyl tRNA synthetase inhibitors, for the treatment of tuberculosis

Authors

Susan H. Davis[1], Michael Mathieson[1], Kirsteen I. Buchanan[1], Alice Dawson[1], Alasdair Smith[1], Mattia Cocco[1], Fabio K. Tamaki[1], John M. Post[1], Beatriz Baragaña[1], Chimed Jansen[1], Michael Kiczun[1], Fabio Zuccotto[1], Gavin Wood[1], Paul Scullion[1], Peter C. Ray[1], Ola Epemolu[1], Eva Maria Lopez-Román[3], Laura Guijarro López[3], Curtis A. Engelhart[2] Jia Kim[2], Paula A. Pino[2], Dirk Schnappinger[2], Kevin D. Read[1], Lourdes Encinas[3], Robert H Bates[3], Paul G Wyatt[1], Simon R. Green[1], and Laura A.T. Cleghorn*[1]

Affiliations

[1]Drug Discovery Unit, Division of Biological Chemistry and Drug Discovery, College of Life Sciences, University of Dundee, Dundee, DD1 5EH, UK

[2]Dept. of Microbiology and Immunology, Weill Cornell Medical College, New York, NY 10065, USA

[3]Global Health Medicines R&D, GlaxoSmithKline, Severo Ochoa 2, Tres Cantos, 28760, Madrid, Spain

***Corresponding author**

l.a.t.cleghorn@dundee.ac.uk

ORCHID iD 0000-0001-6218-0092

**Table of Contents**	Page
X-ray crystallographic data collection and refinement statistics	S2
Pharmacokinetic data collected throughout the project	S4
In vivo exposure for the four most advanced compounds	S4
Electron density maps for compounds crystallised in LysRS	S5
In vivo metabolite identification for **11**	S5
Additional co-crystal structures	S6
Surface view of **49** in the ATP binding pocket of LysRS	S6
1H NMR spectra for compounds in manuscript	S7
13C NMR Spectra for compounds progressed to in vivo studies	S27
HRMS for compounds progressed to in vivo studies	S32
Supplementary Methods	S38

Table S1: Crystallography data collection and refinement statistics.

	**10**	**25**	**27**
**Data collection**			
Source	ESRF ID23-1	ESRF ID23-1	Diamond I04-1
Wavelength	0.97625	0.97625	0.91587
Space group	$P4_12_12$	$P4_12_12$	$P4_12_12$
Cell dimensions			
a, c (Å)	85.00, 148.04	83.93, 147.29	83.58, 147.41
Resolution (Å)	2.3 (2.38-2.30)	2.4 (2.49-2.40)*	2.4 (2.49-2.40)
R_{merge}	0.117 (1.218)	0.113 (2.311)	0.254 (5.109)
$I / \sigma I$	10.7 (2.0)	8.5 (0.6)	6.5 (0.7)
CC1/2	0.999 0.960)	0.999 (0.508)	0.997 (0.597)
Completeness (%)	92.1 (99.9)**	96.4 (99.0)**	100 (100)
Redundancy	10.0 (11.2)	7.6 (7.7)	14.4 (14.1)
**Refinement**			
Resolution (Å)	2.30	2.40	2.40
No. reflections	21736	19448	20063
R_{work} / R_{free}	25.5 / 31.1	25.0 / 32.0	23.2 / 30.8
No. atoms			
Protein	3646	3624	3595
Ligand / lysine	20 / 10	21 / 10	20 / 10
Water	11	26	58
B-factors			
Protein	75.8	91.7	80.8
Ligand / Lysine	54.2 / 64.4	69.0 / 82.0	60.0 / 64.0
Water	52.0	61.3	58.7
R.m.s. deviations			
Bond lengths (Å)	0.0063	0.0115	0.0053
Bond angles (°)	1.4472	2.0607	1.5189
PDB codes	9qea	9qei	9qbr

*Values in parentheses are for highest-resolution shell.
** completeness is lower due to significant ice ring

Table S1 (cont.): Crystallography data collection and refinement statistics.

	32	37	42
**Data collection**			
Source	Inhouse	Diamond I04-1	Inhouse
Wavelength	1.54178	0.91188	1.54178
Space group	$P4_12_12$	$P4_12_12$	$P4_12_12$
Cell dimensions			
a, c (Å)	83.70, 147.69	83.67, 147.26	83.98, 147.32
Resolution (Å)	2.6 (2.71-2.60)	2.28 (2.32-2.28)	2.6 (2.72-2.60)*
R_{merge}	0.360 (2.404)	0.142 (1.702)	0.158 (0.522)
$I / \sigma I$	4.8 (0.8)	11.1 (0.9)	8.5 (2.3)
CC1/2	0.988 (0.589)	0.991(0.894)	0.988 (0.972)
Completeness (%)	99.7 (98.1)	100 (100)	99.7 (97.5)
Redundancy	11.2 (9.6)	12.7 (12.8)	13.8 (11.4)
**Refinement**			
Resolution (Å)	2.60	2.28	2.60
No. reflections	15953	23355	15966
R_{work} / R_{free}	26.0 / 31.8	21.4 / 27.7	23.2 / 29.2
No. atoms			
Protein	3654	3591	3595
Ligand / lysine	21 / 10	21 / 10	27 / 10
Water	12	82	31
B-factors			
Protein	65.7	69.8	56.3
Ligand / Lysine	42.1 / 46.6	52.2 / 48.7	43.3 / 40.7
Water	37.2	52.2	34.1
R.m.s. deviations			
Bond lengths (Å)	0.0044	0.0082	0.0036
Bond angles (°)	1.2122	1.5664	1.0064
PDB codes	9qdj	9qc3	9qc4

*Values in parentheses are for highest-resolution shell.

Table S2: Pharmacokinetic data collected throughout the study.

	8	10	11	25	34	37	49
C_{max} (ng/mL)	1,400	5,300	4,800	4,260	339	2,670	3700
AUC (µg/mL.min)	110	220	270	400	68	450	500
Cl (mL/min/kg)	28	19	16	23	29	22	15
$T_{1/2}$ (h)	0.3	0.3	0.3	0.4	1	2	1.4
V_d (L/kg)	0.7	0.4	0.4	0.7	4	2.2	1.0
F (%)	31	43	43	91	16	100	74

Comparative murine pharmacokinetic parameters obtained across the SAR program

Table S3: *In vivo* exposure for four most advanced compounds.

	46	47	48	49
C_{max} (ng/mL)	118,000	25,300	50,500	35,100
AUC (µg/mL.min)	169	36	100	181

Total blood concentrations taken at four time-points on first day of dosing from individual infected mice.

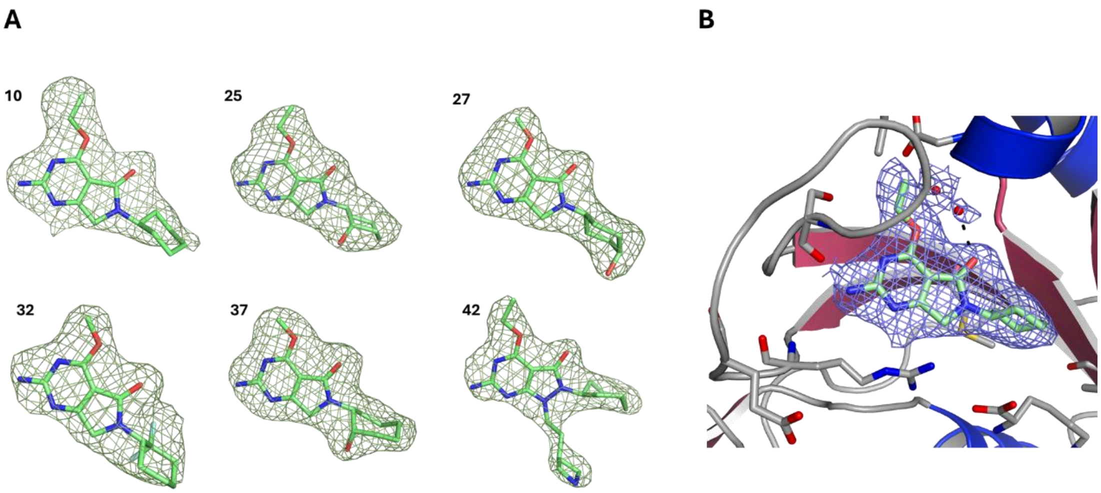

Figure S1: Crystallographic electron density maps supporting ligand binding.
A. The difference density omit map (chicken wire) for each ligand, calculated with (Fo-Fc), αc coefficients and contoured at the 3 σ level. Fo represents the observed structure factors, Fc the calculated structure factors and αc the calculated phases. The atomic co-ordinates depicted in this figure did not contribute to Fc or αc. Ligands are shown with atomic positions coloured C lime, N blue, O red, F pale green. **B.** The same view as **Figure 1A** but showing the conventional (2Fo-Fc) map for compound **10** and its surrounding waters, contoured at the 1 σ level. Figure prepared using PyMol: The PyMOL Molecular Graphics System, Version 2.5.5 Schrödinger, LLC.

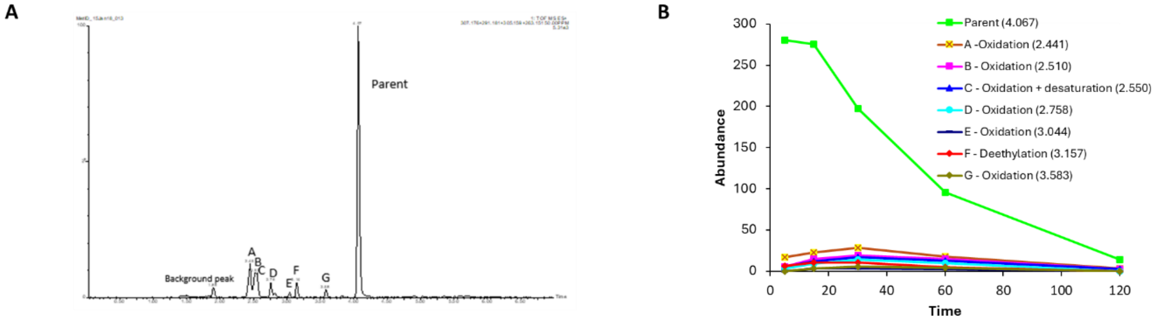

C

Peak ID	Tentative Met. Id.	Proposed [M+H]+ ([M-H]-)	Ret. Time - min acidic (basic)	% Peak Area	Product Ions - positive (negative)
Parent (P)	Parent	291	4.07	67	55,97,98,125,150,167,221,263,291
A	P + O	307	2.44	10	67,95,125,150,167,195
B	P + O	307	2.51	7	67,95,125,150,167,195,261,279
C	P + O - 2H	305	2.55	6	93,111,125,167,195,200
D	P + O	307	2.76	5	95,125,150,167,195,200,000
E	P + O	307	3.04	1	No data
F	P - C2H4	263	3.16	4	125,167,263
G	P + O	307	3.58	2	125,167

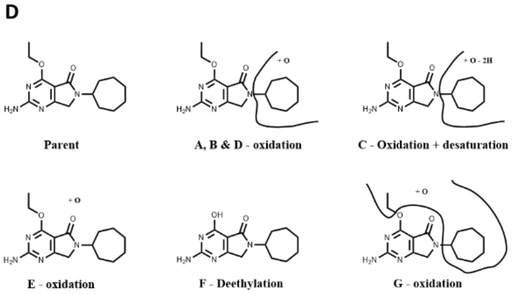

Figure S2: *In vivo* metabolite identification for 11.
A. Overlaid extracted ion chromatogram (EIC) of parent compound and identified metabolites (A to G). **B.** Change in ion abundance with time for identified mass spectrometer signals for parent compound and identified metabolites. **C.** Summary table showing parent compound and identified metabolites with their precursor ion masses, retention time, % peak area (% of total peak area of the proposed [M+H]+ ions at 30 min time point) and MS2 fragment ions. **D.** Proposed location of identified metabolic transformations based upon the observed MS2 fragment ions.

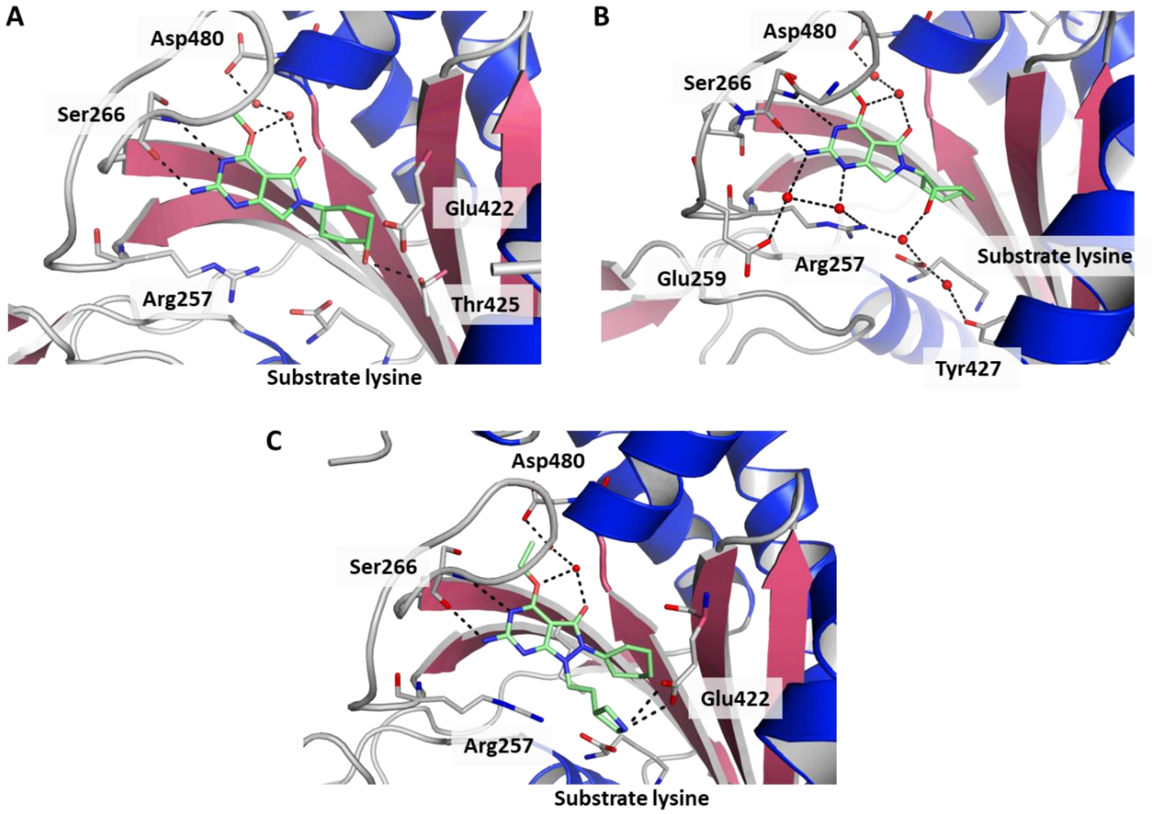

Figure S3: Additional co-crystal structures.

The ligands are shown with mint green carbon atoms, blue nitrogen atoms and red oxygen atoms. Key water molecules are shown as red spheres and hydrogen bonds by dashed lines. Key residues involved with binding are shown. **A. 27** (PDB 9qbr); **B. 37** (PDB 9qc3) and **C. 42** (PDB 9qc4). Figures prepared using PyMol: The PyMOL Molecular Graphics System, Version 2.5.5 Schrödinger, LLC.

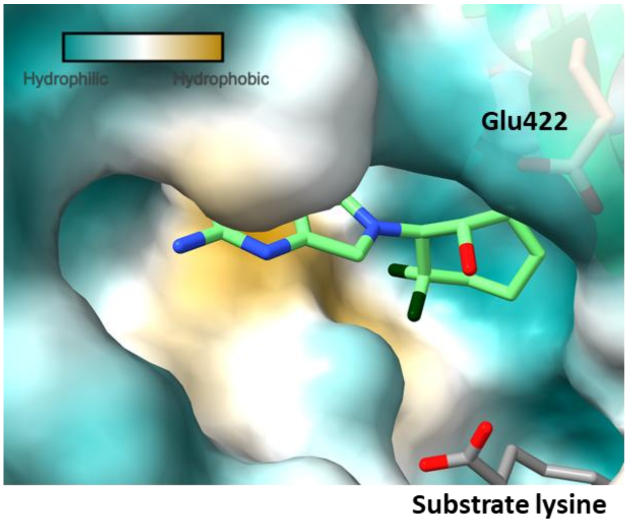

Figure S4: Surface view of 49 in the ATP binding pocket of LysRS.

Figure prepared as Figure 3 in the main article. The polar hydroxyl group of **49** sits in the more hydrophilic part of the pocket pointing towards Glu422. While the hydrophobic di-fluoro group sits in the hydrophobic area formed by the side chain of Met271 (yellow surface).

1H NMR spectra for compounds in manuscript.

2

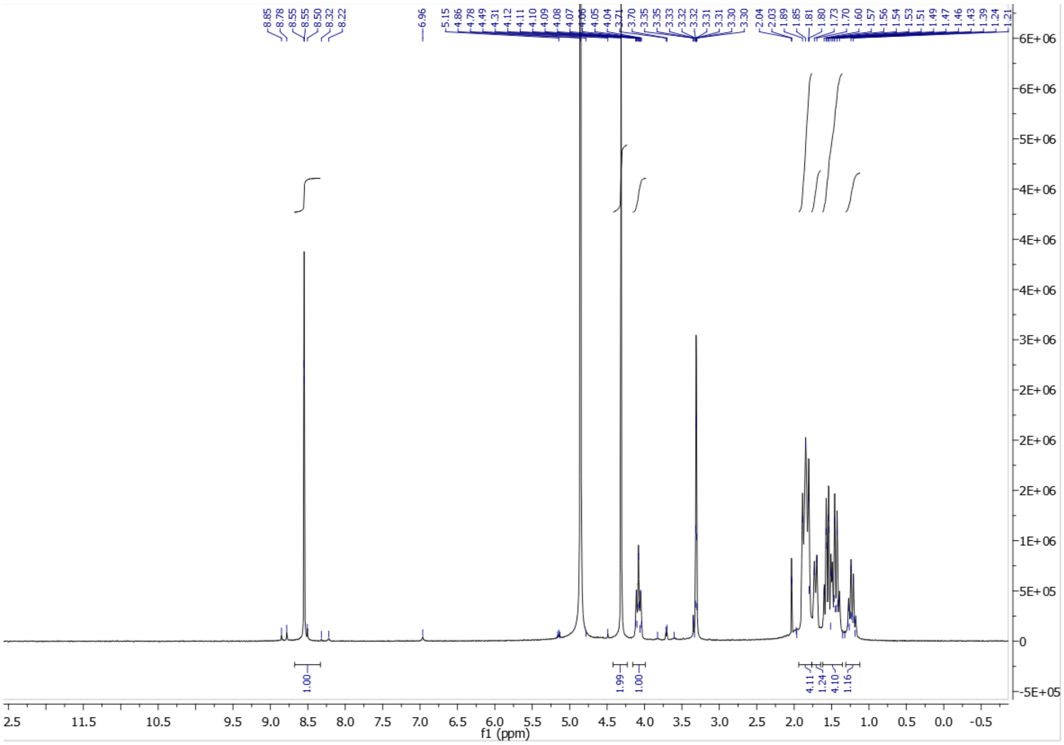

3

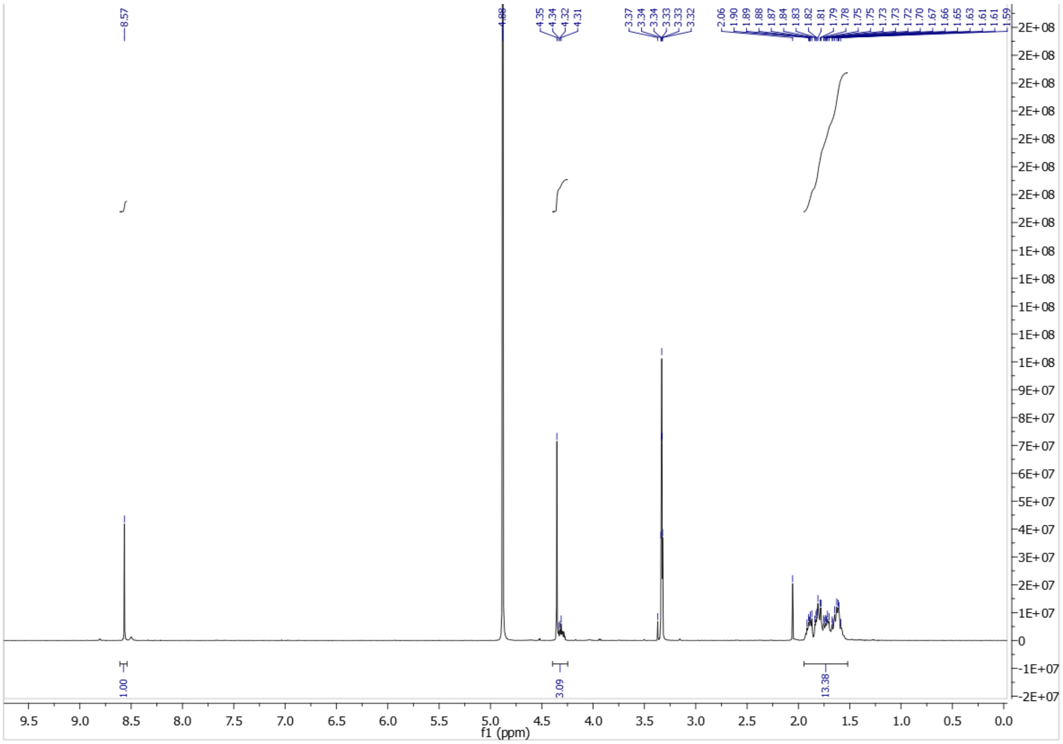

4

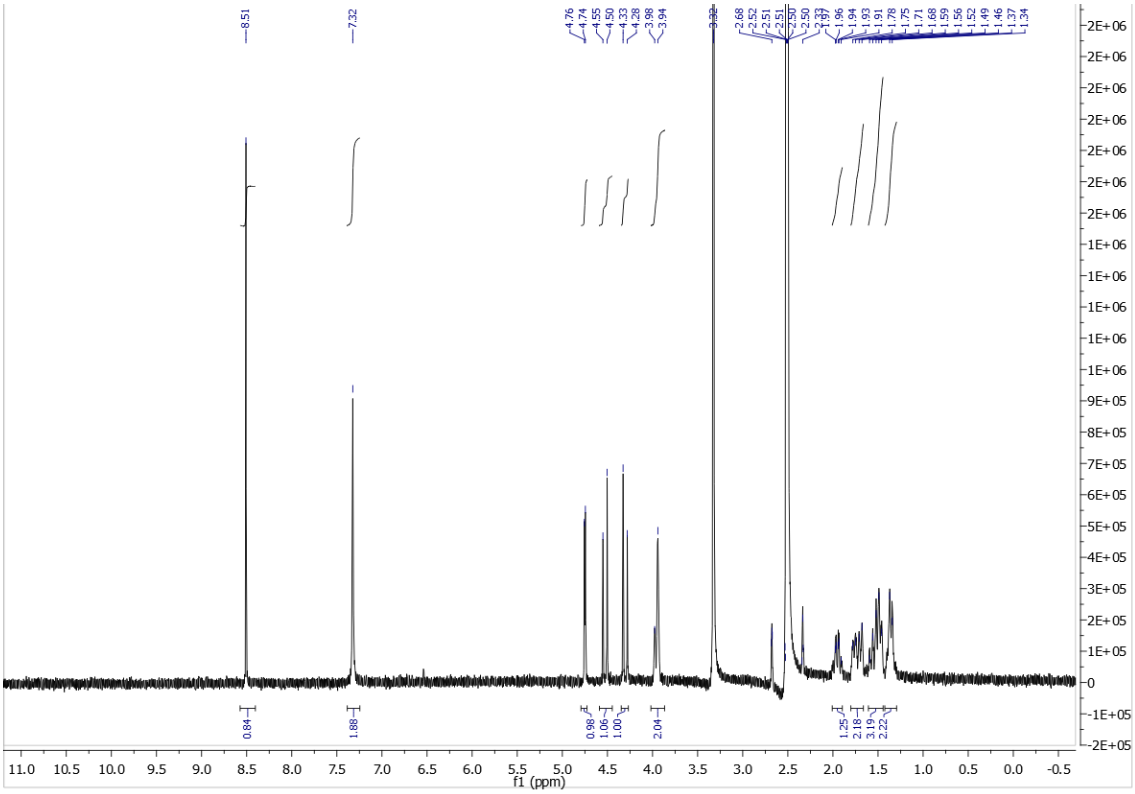

8.51
7.32
4.76 4.74 4.55 4.50 4.33 4.28 3.98 3.94
3.32
2.68 2.52 2.51 2.51 2.50 2.50 2.33 1.97 1.96 1.94 1.93 1.91 1.78 1.75 1.71 1.68 1.59 1.56 1.52 1.49 1.46 1.37 1.34
0.84
1.88
0.98 1.06 1.00
2.04
1.25 2.18 3.19 2.22
f1 (ppm)

5

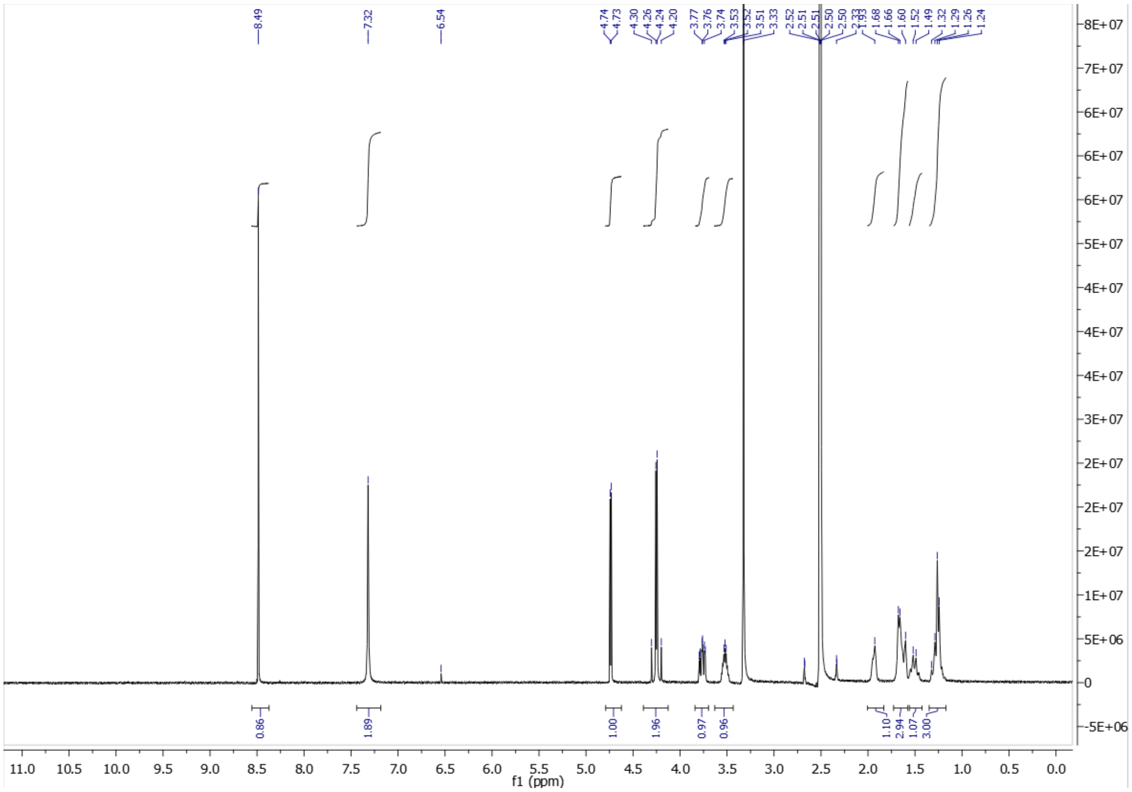

8.49
7.32
6.54
4.74 4.73 4.30 4.26 4.24 4.20 3.77 3.76 3.74 3.53 3.52 3.51 3.33 2.52 2.51 2.51 2.50 2.33 1.93 1.68 1.66 1.60 1.52 1.49 1.32 1.29 1.26 1.24
0.86
1.89
1.00
1.96
0.97
0.96
1.10 2.94 1.07 3.00
f1 (ppm)

6

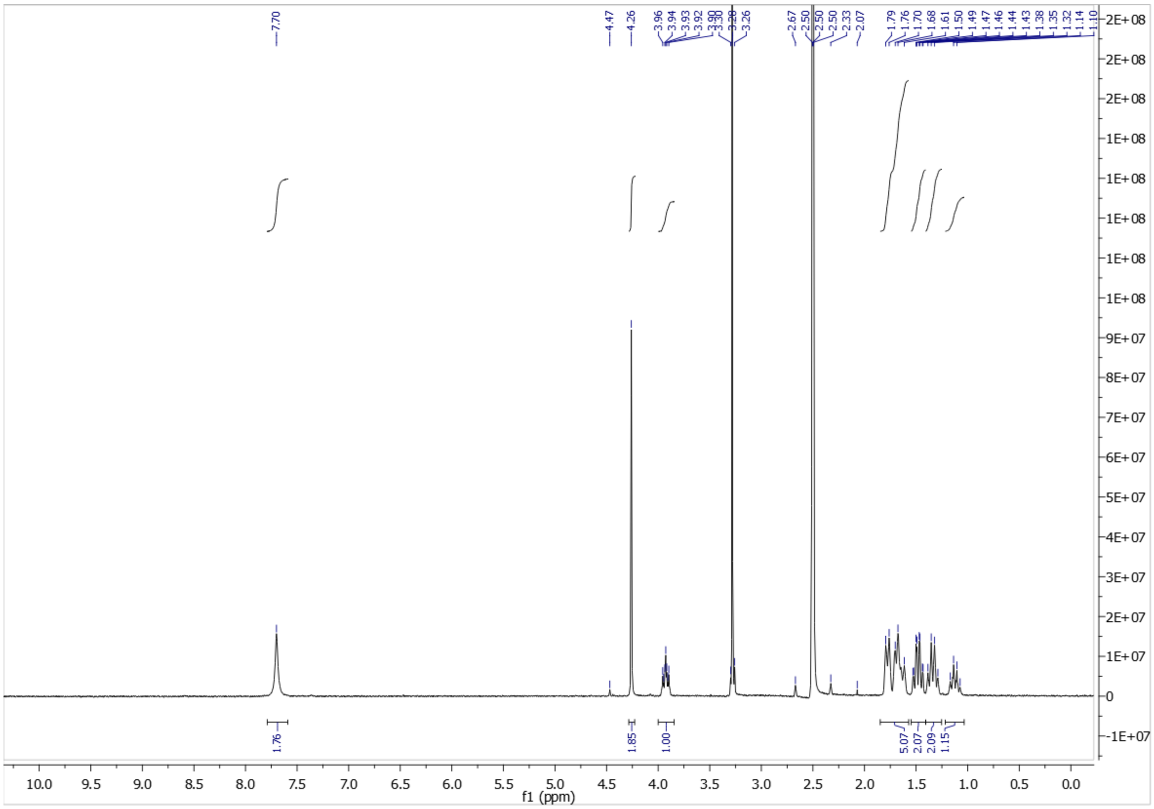

7

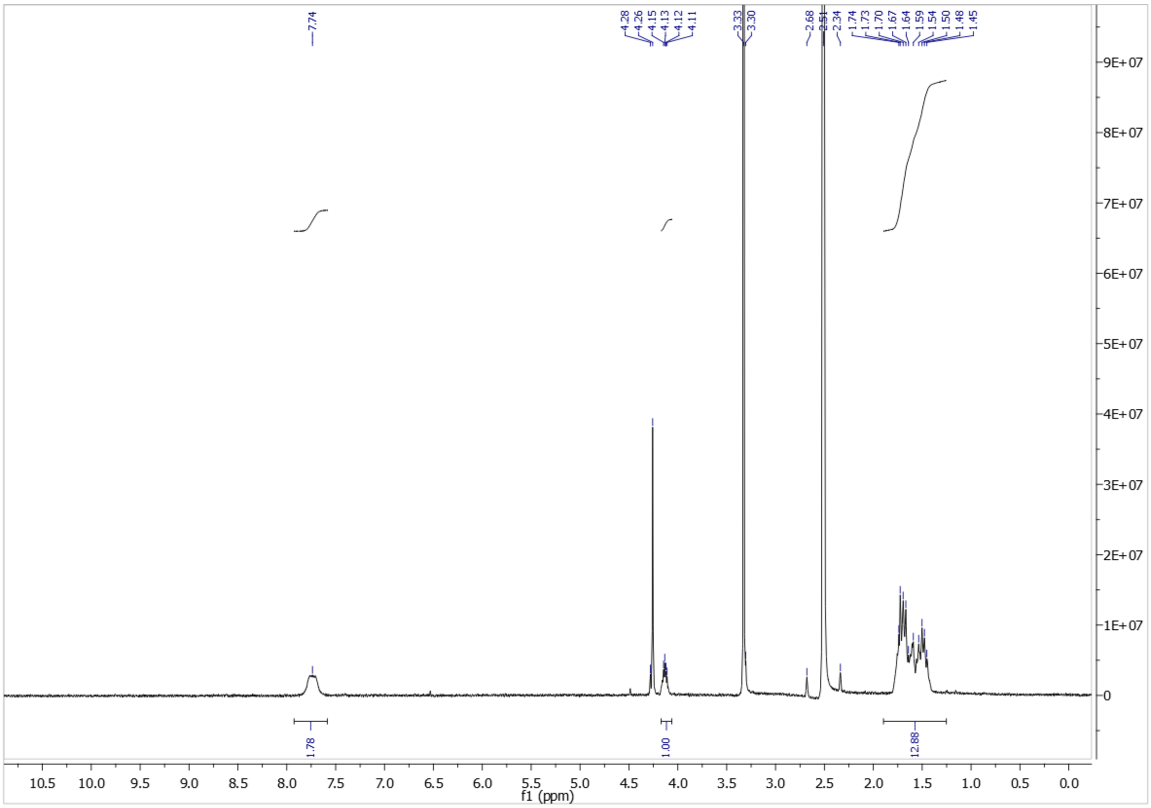

8

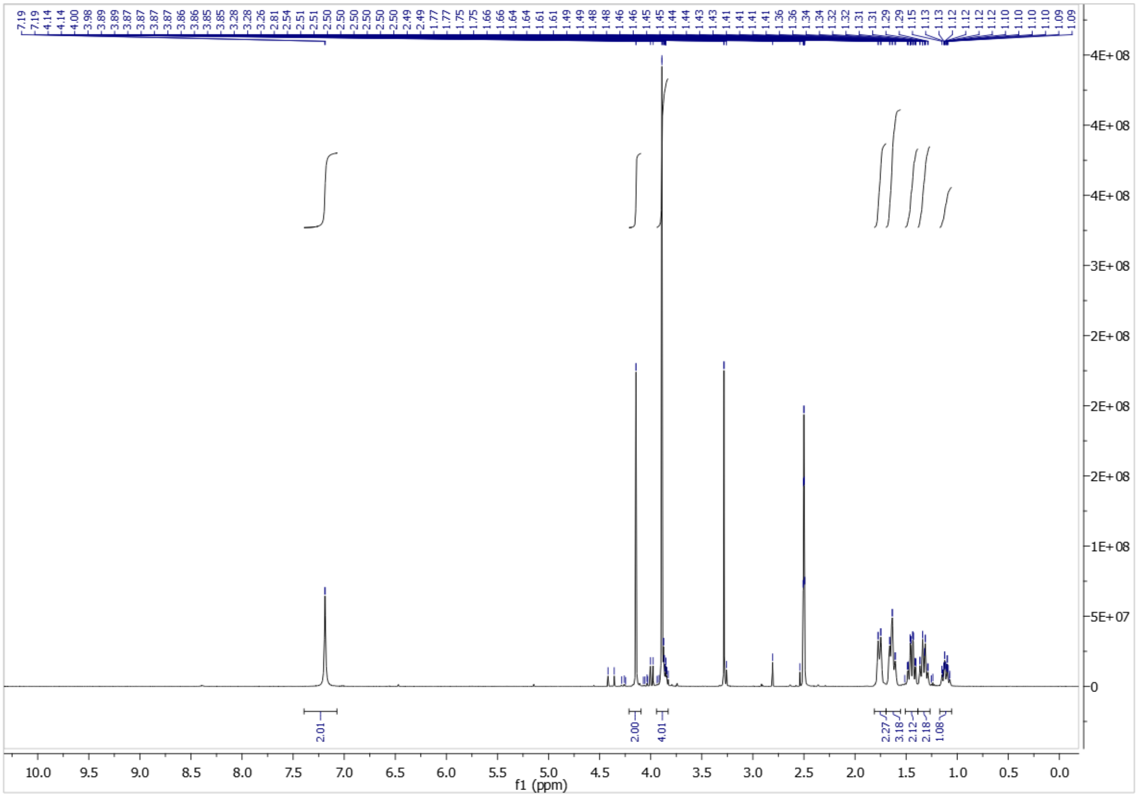

9

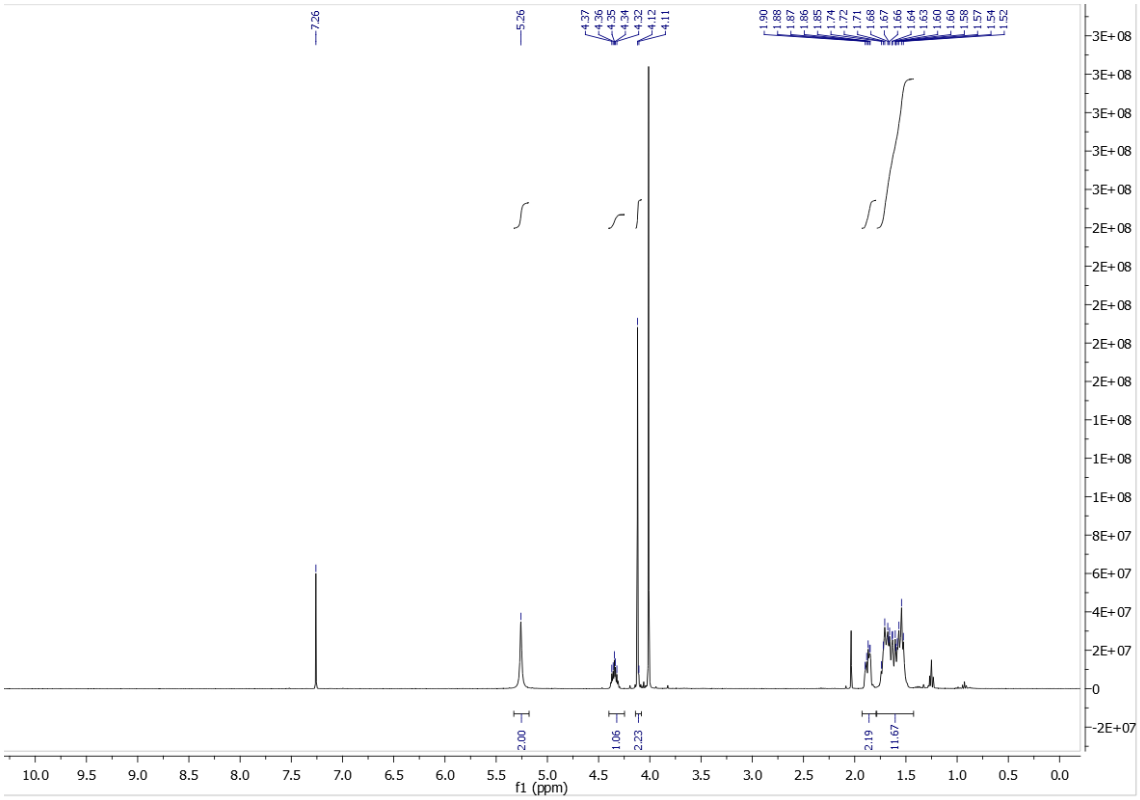

12

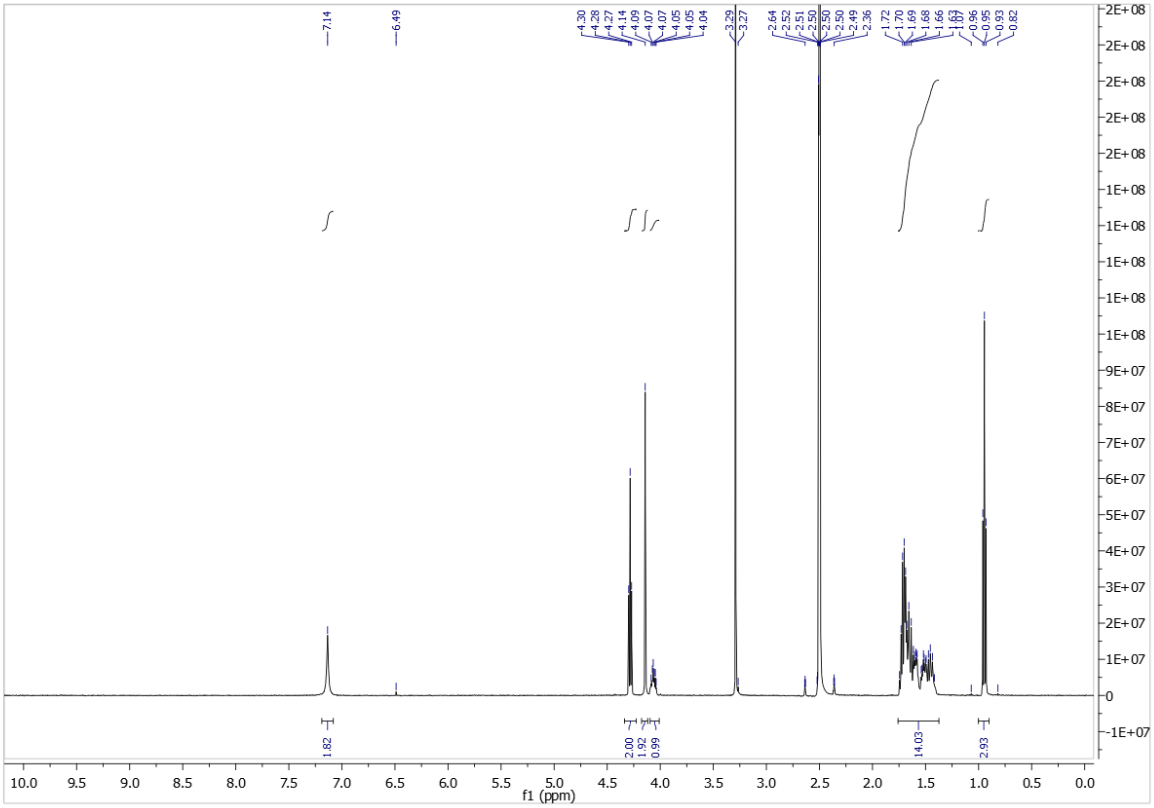

13

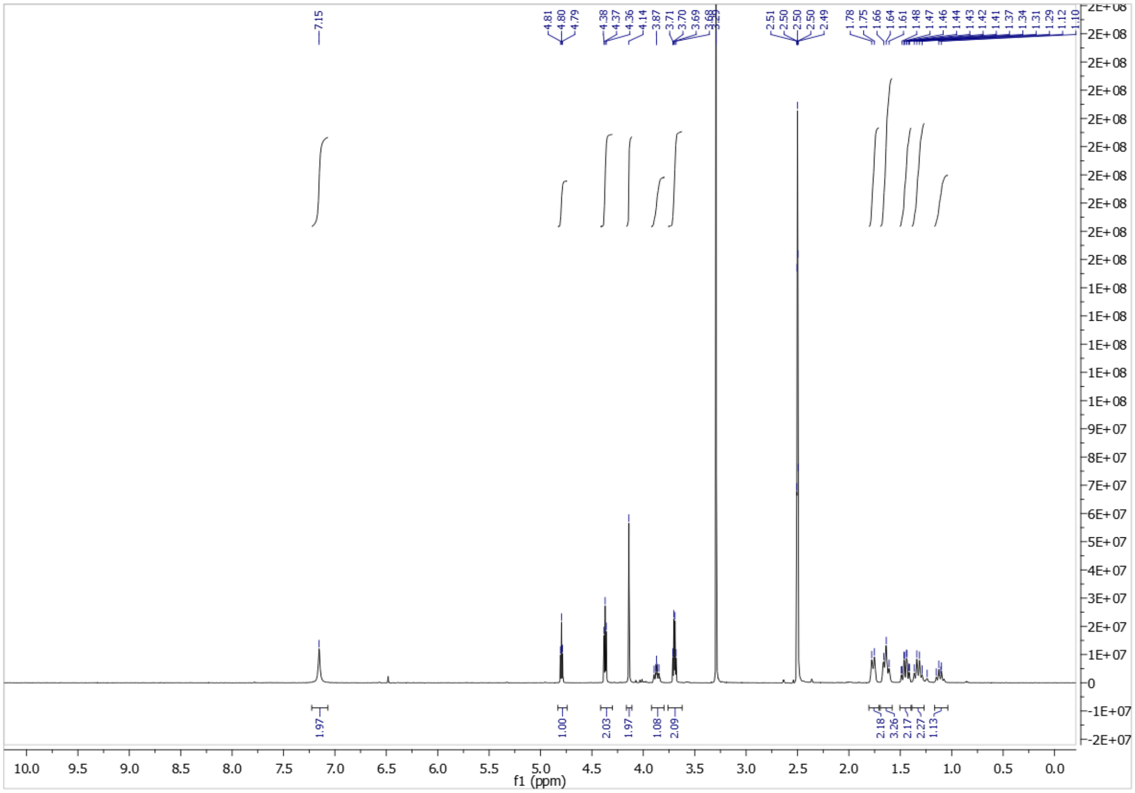

14

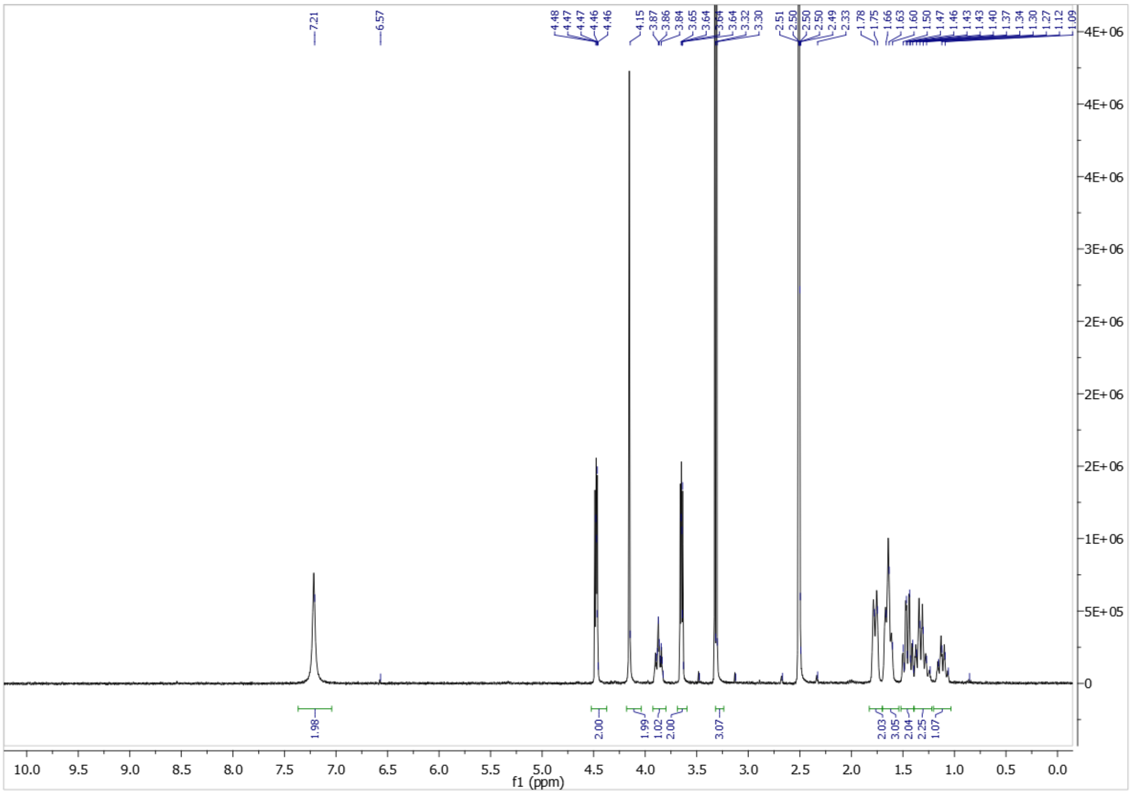

15

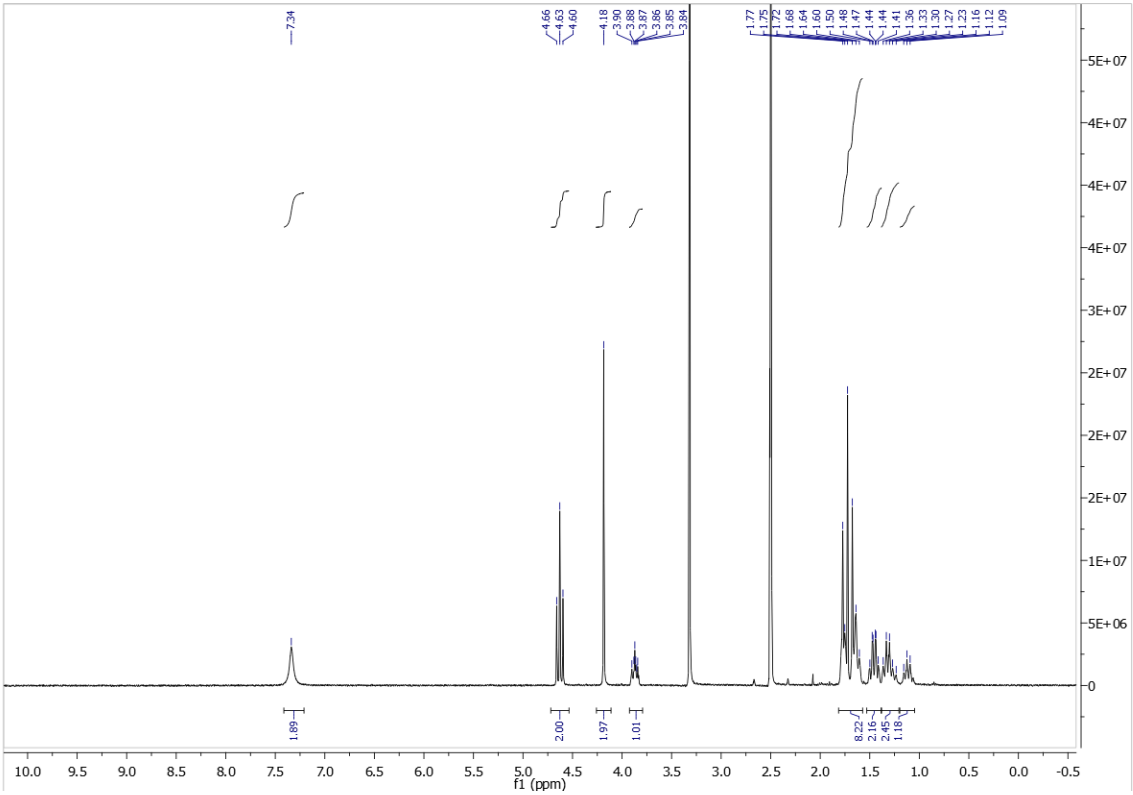

16

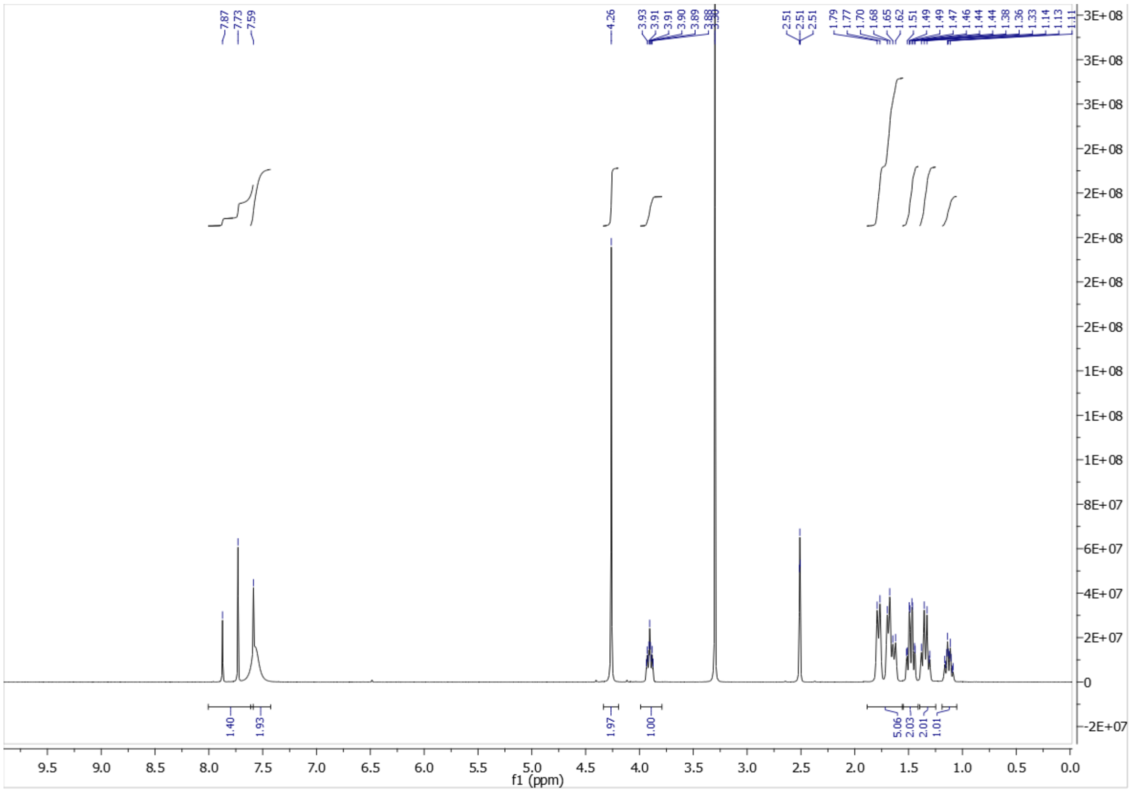
7.87
7.73
7.59
4.26
3.93
3.91
3.91
3.90
3.89
3.88
2.51
2.51
2.51
1.79
1.77
1.70
1.68
1.65
1.62
1.51
1.49
1.49
1.47
1.46
1.44
1.44
1.38
1.36
1.33
1.14
1.13
1.11
1.40
1.93
1.97
1.00
5.06
2.03
2.01
1.01
f1 (ppm)

17

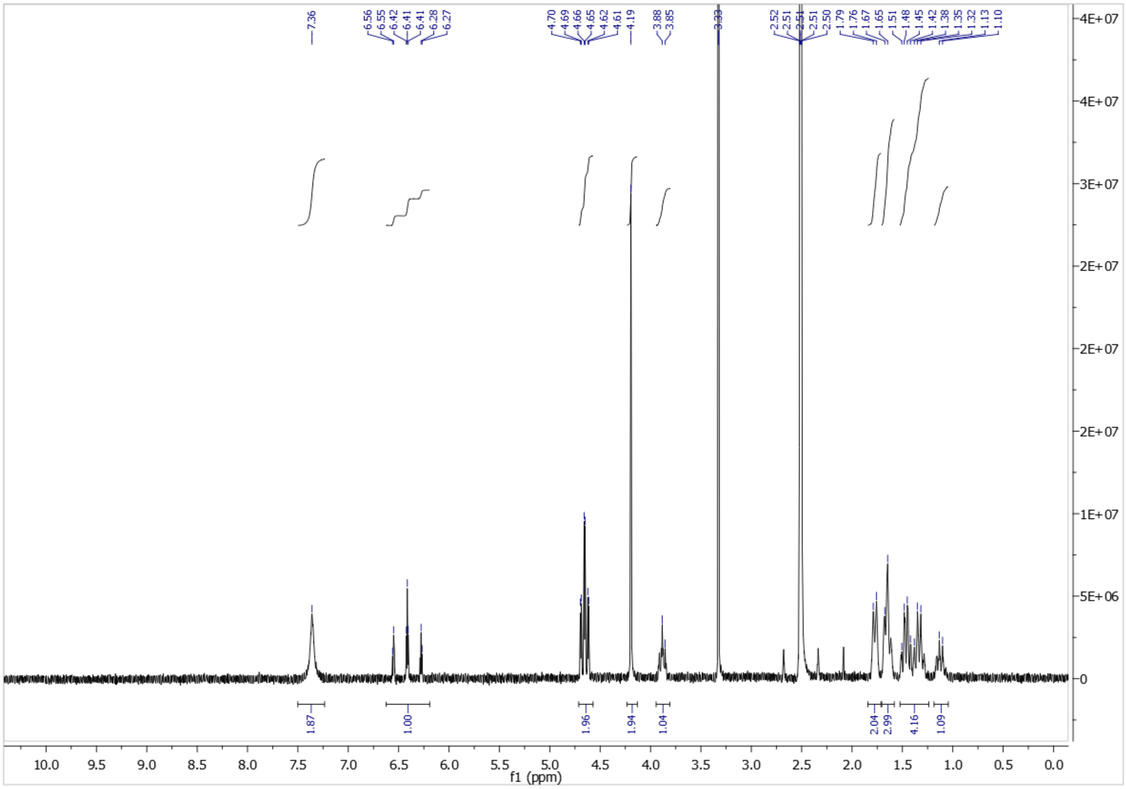
7.36
6.56
6.55
6.42
6.41
6.41
6.28
6.27
4.70
4.69
4.66
4.65
4.62
4.61
4.19
3.88
3.85
3.33
2.52
2.51
2.51
2.51
2.50
1.79
1.76
1.67
1.65
1.51
1.48
1.45
1.42
1.38
1.35
1.32
1.13
1.10
1.87
1.00
1.96
1.94
1.04
2.04
2.99
4.16
1.09
f1 (ppm)

18

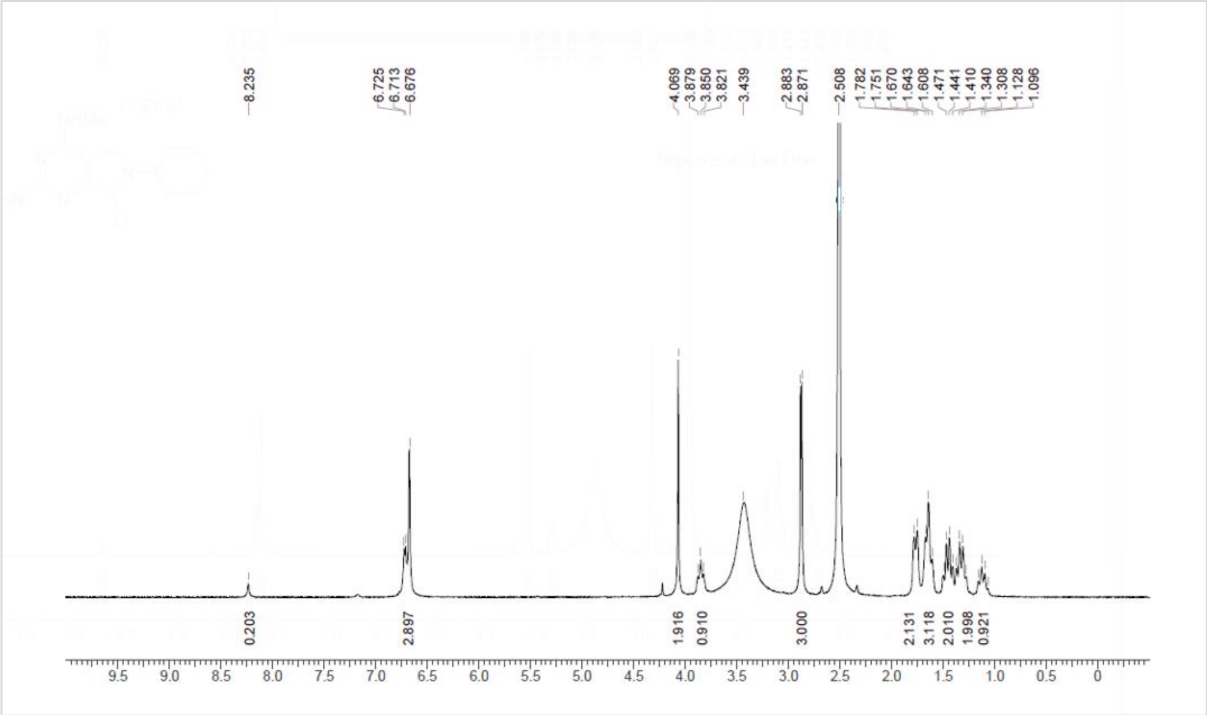

19

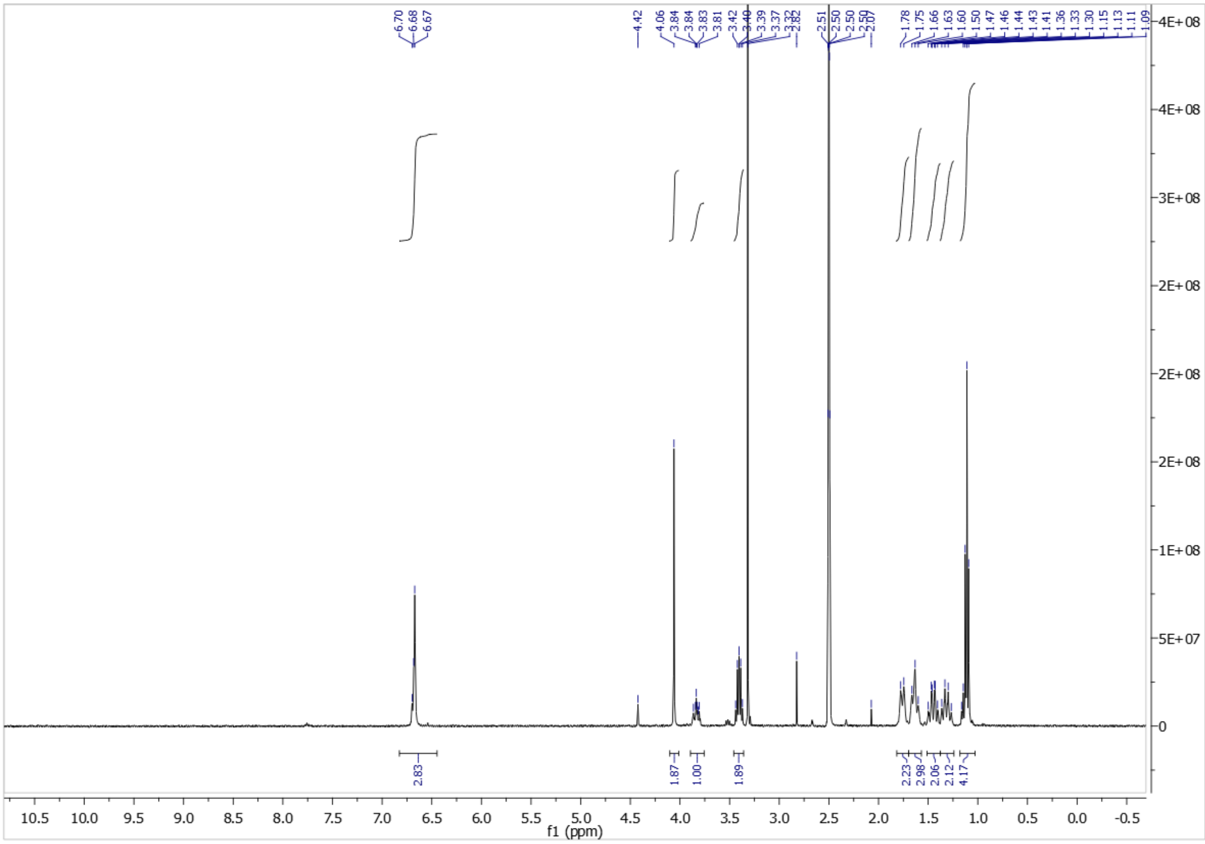

20

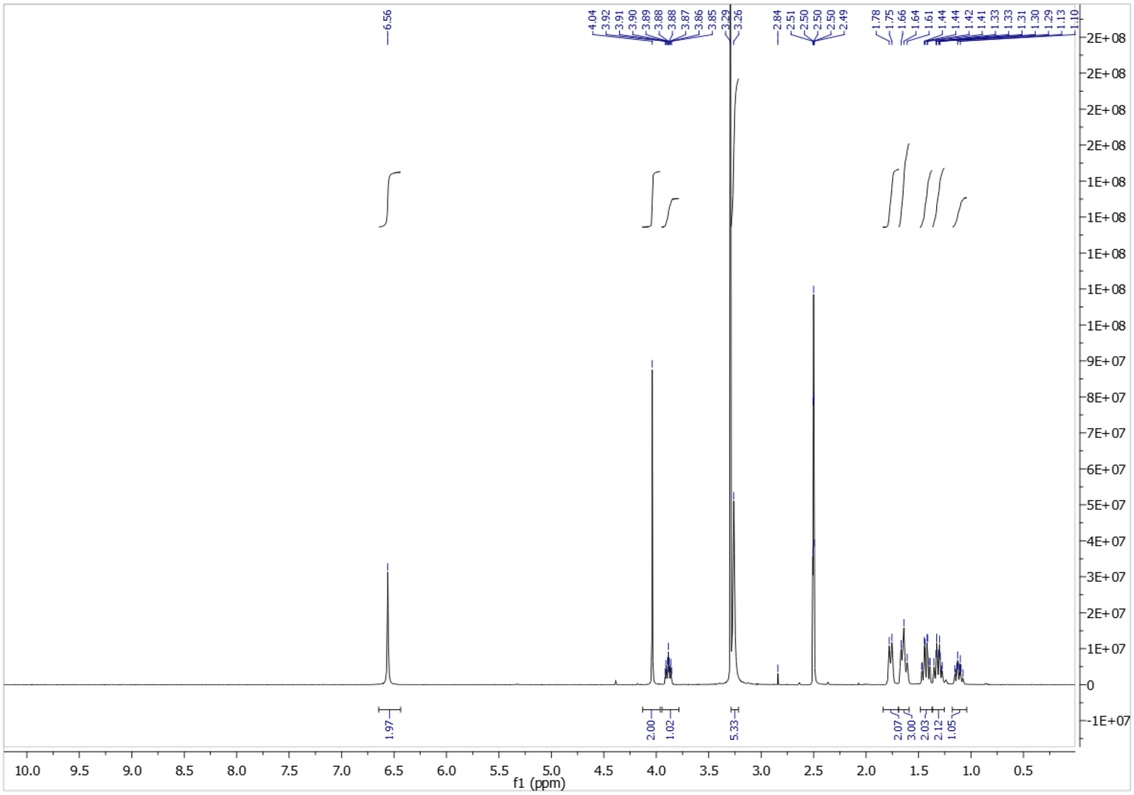

21

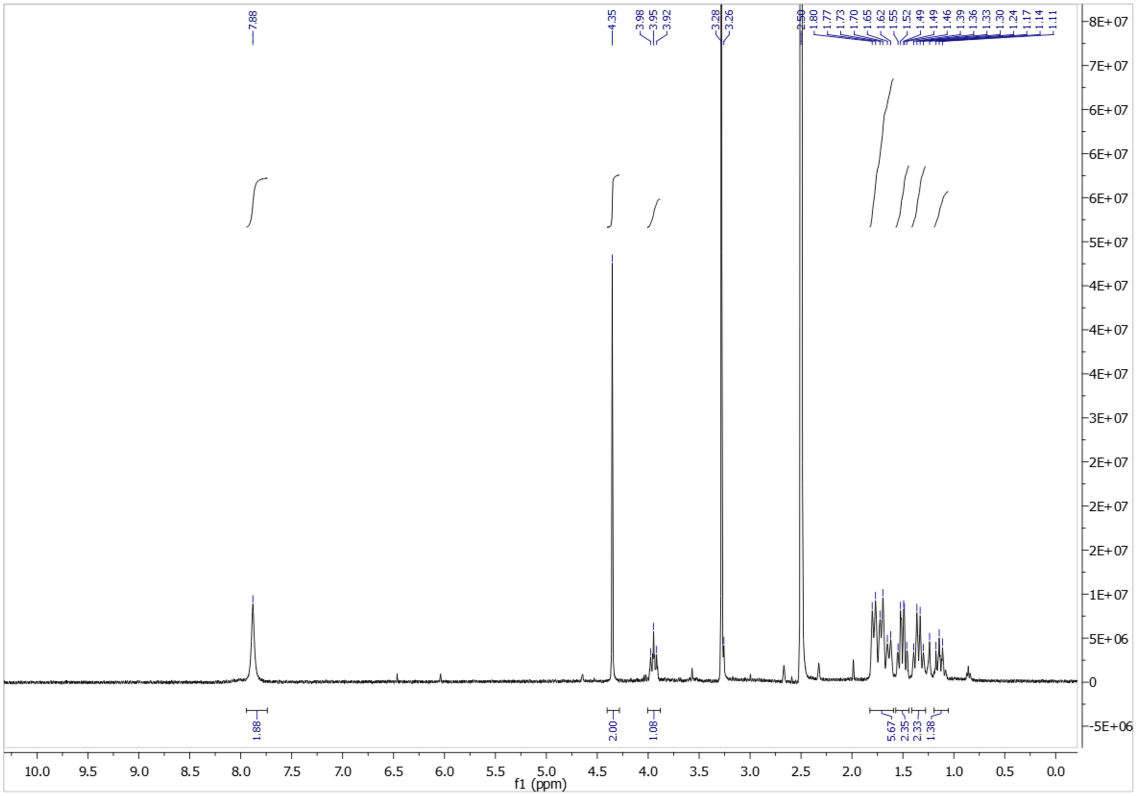

22

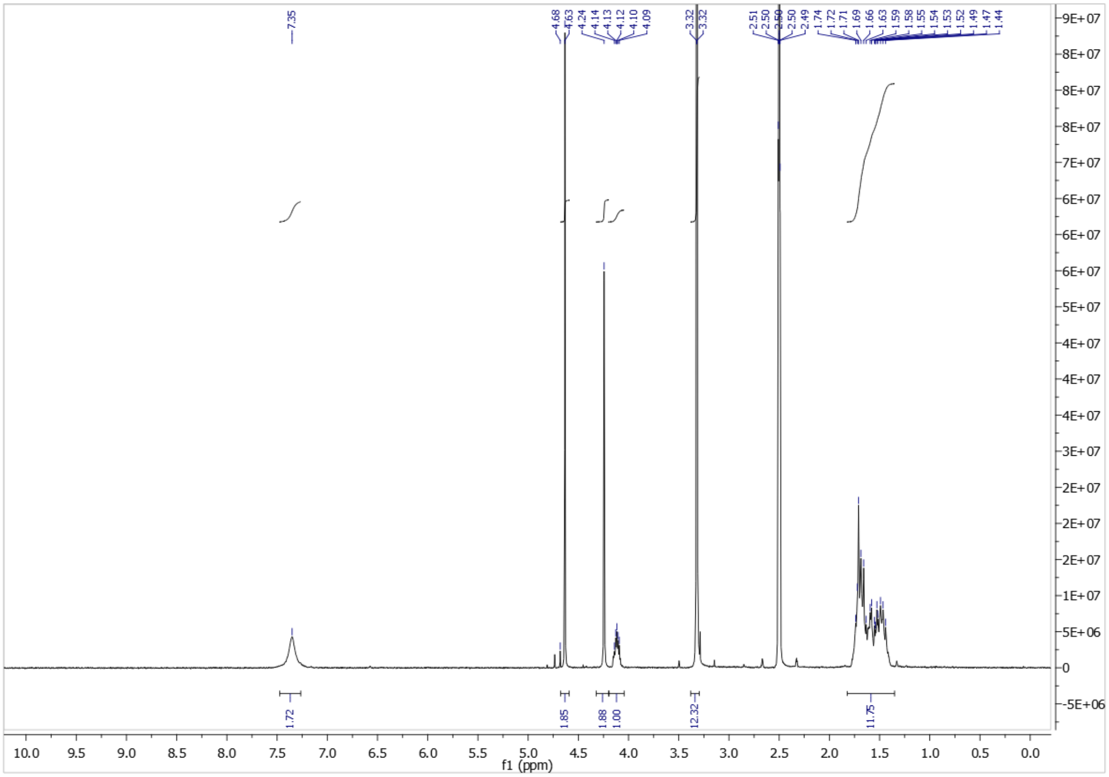
7.35
4.68 4.63 4.24 4.14 4.13 4.12 4.10 4.09
3.32 3.32
2.51 2.50 2.50 2.50 2.49 1.74 1.72 1.71 1.69 1.66 1.63 1.59 1.58 1.55 1.54 1.53 1.52 1.49 1.47 1.44
1.72 1.85 1.88 1.00 12.32 11.75
f1 (ppm)

23

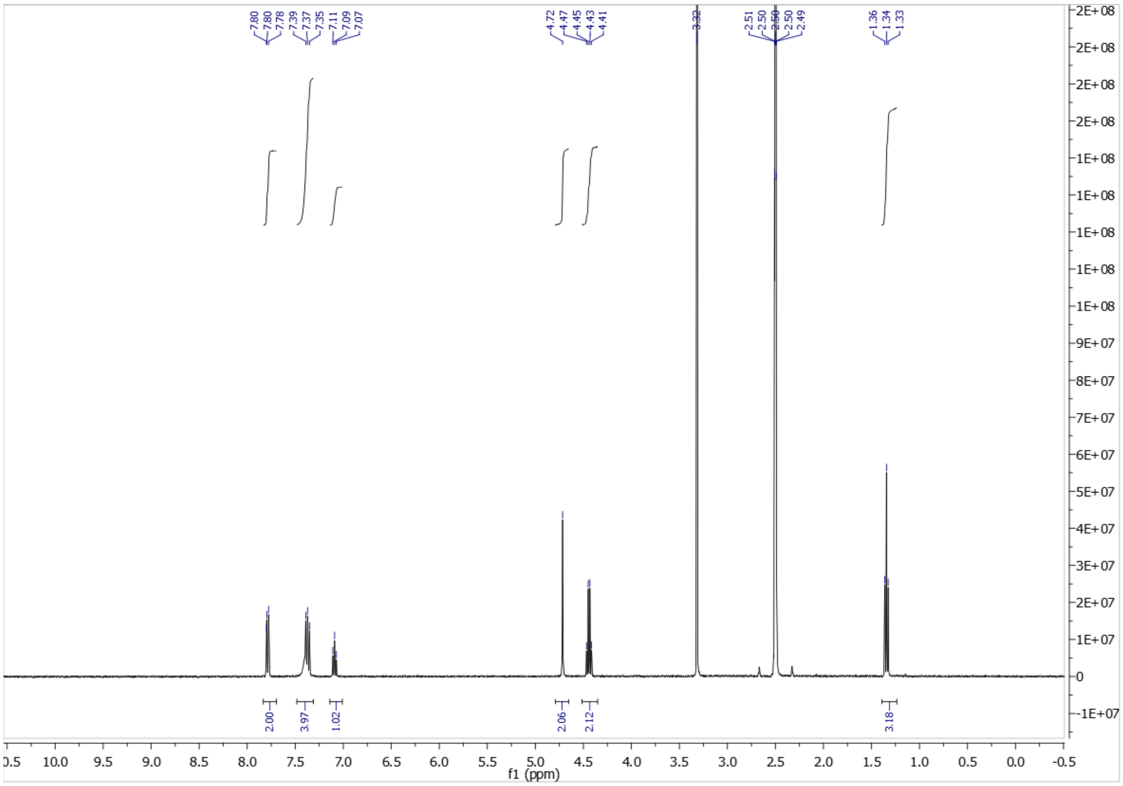
7.80 7.80 7.78 7.39 7.37 7.35 7.11 7.09 7.07
4.72 4.47 4.45 4.43 4.41
3.32
2.51 2.50 2.50 2.50 2.49
1.36 1.34 1.33
2.00 3.97 1.02 2.06 2.12 3.18
f1 (ppm)

26

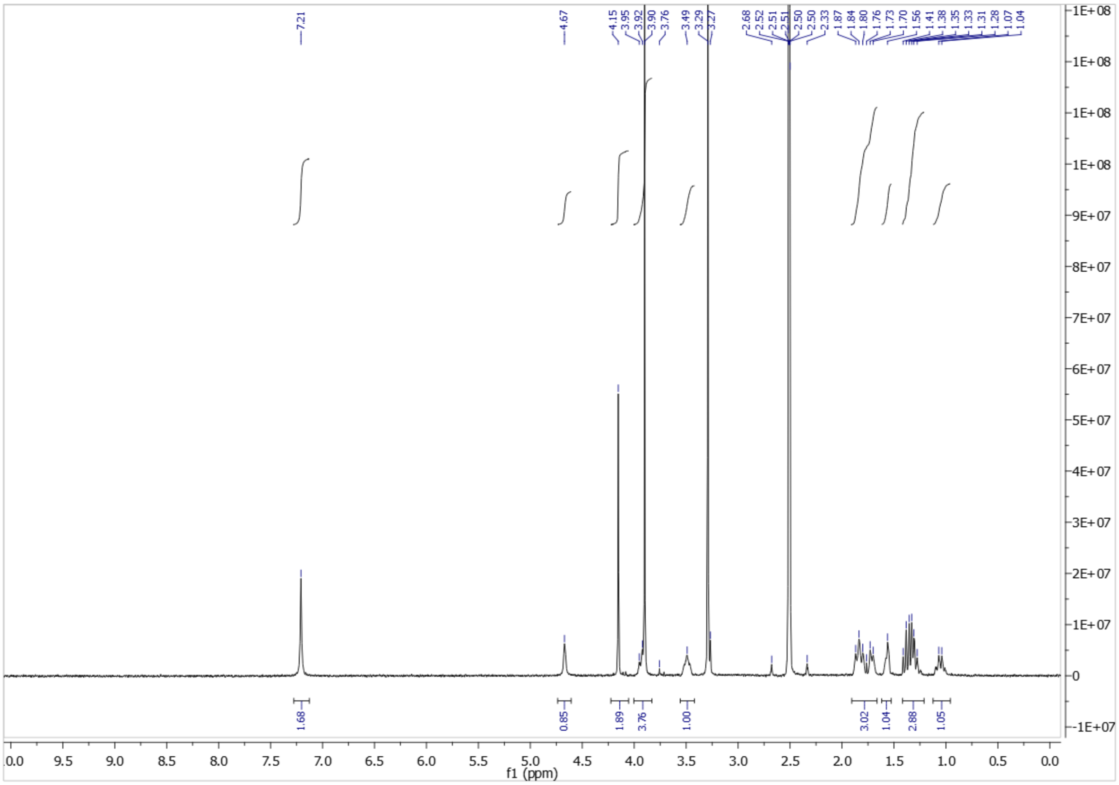

7.21
4.67
4.15
3.95
3.92
3.90
3.76
3.49
3.29
3.27
2.68
2.52
2.51
2.51
2.50
2.50
2.33
1.87
1.84
1.80
1.76
1.73
1.70
1.56
1.41
1.38
1.35
1.33
1.31
1.28
1.07
1.04
1.68
0.85
1.89
3.76
1.00
3.02
1.04
2.88
1.05
f1 (ppm)

27

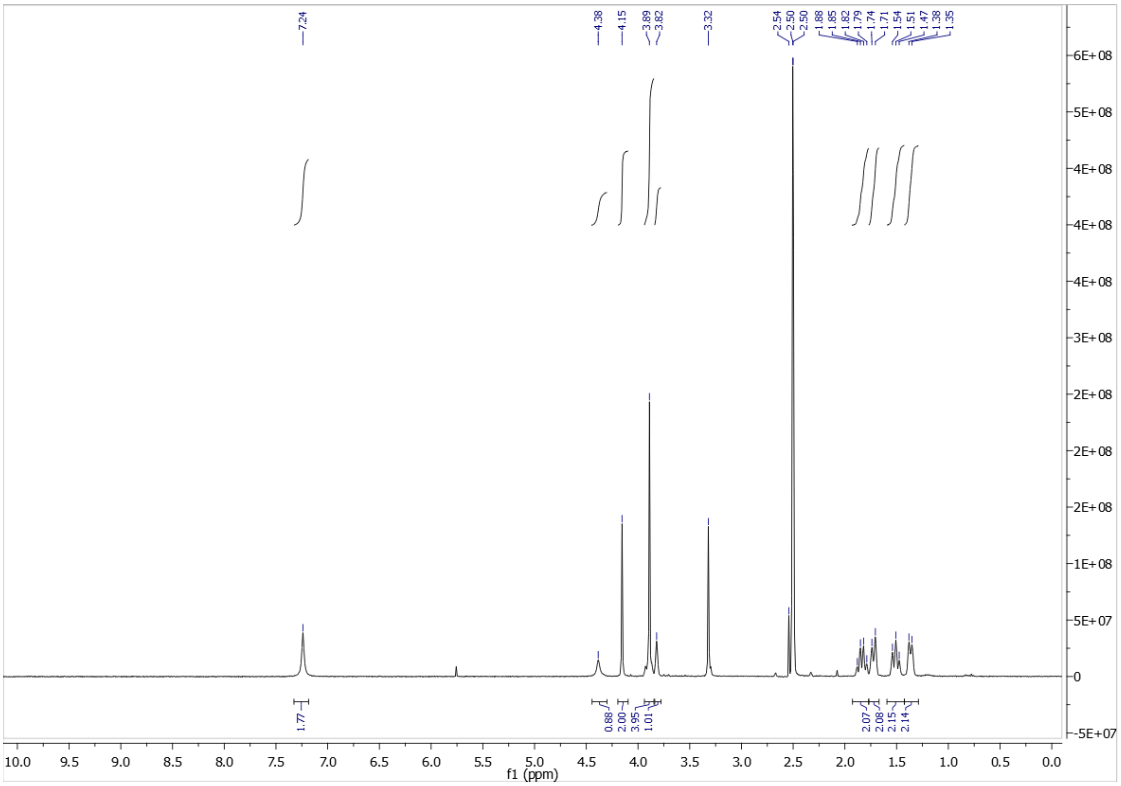

7.24
4.38
4.15
3.89
3.82
3.32
2.54
2.50
2.50
1.88
1.85
1.82
1.79
1.74
1.71
1.54
1.51
1.47
1.38
1.35
1.77
0.88
2.00
3.95
1.01
2.07
2.08
2.15
2.14
f1 (ppm)

28

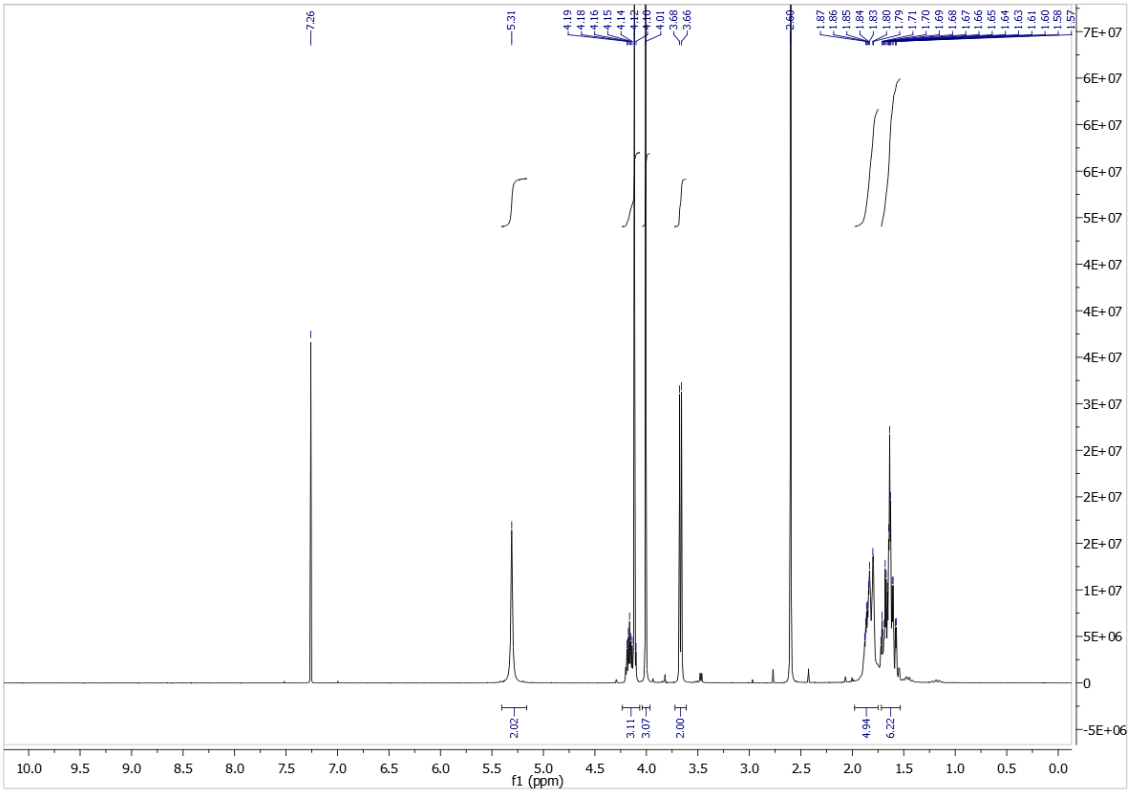

29

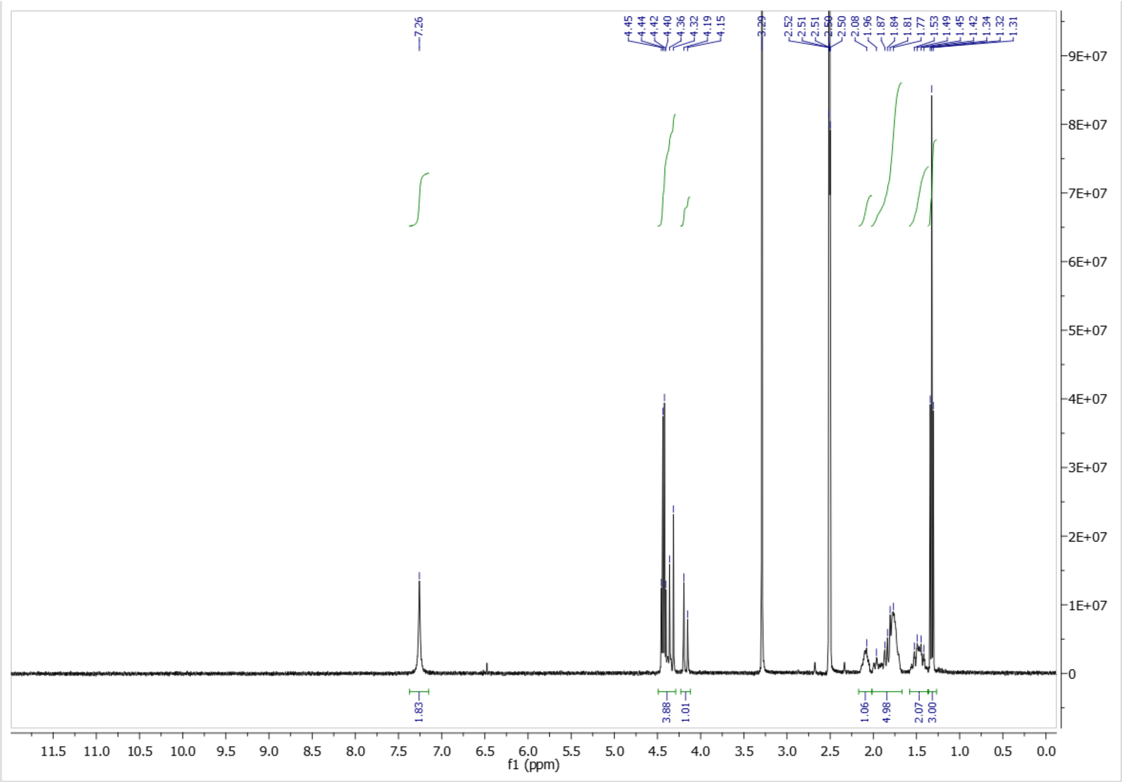

30

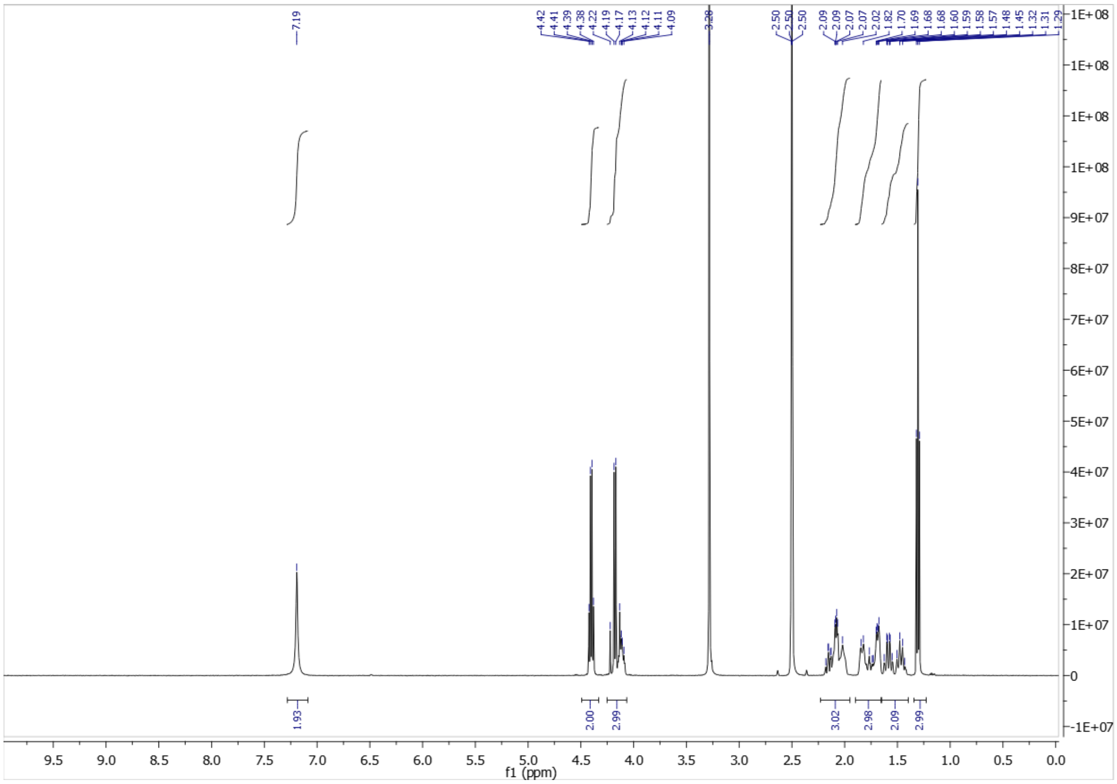

31

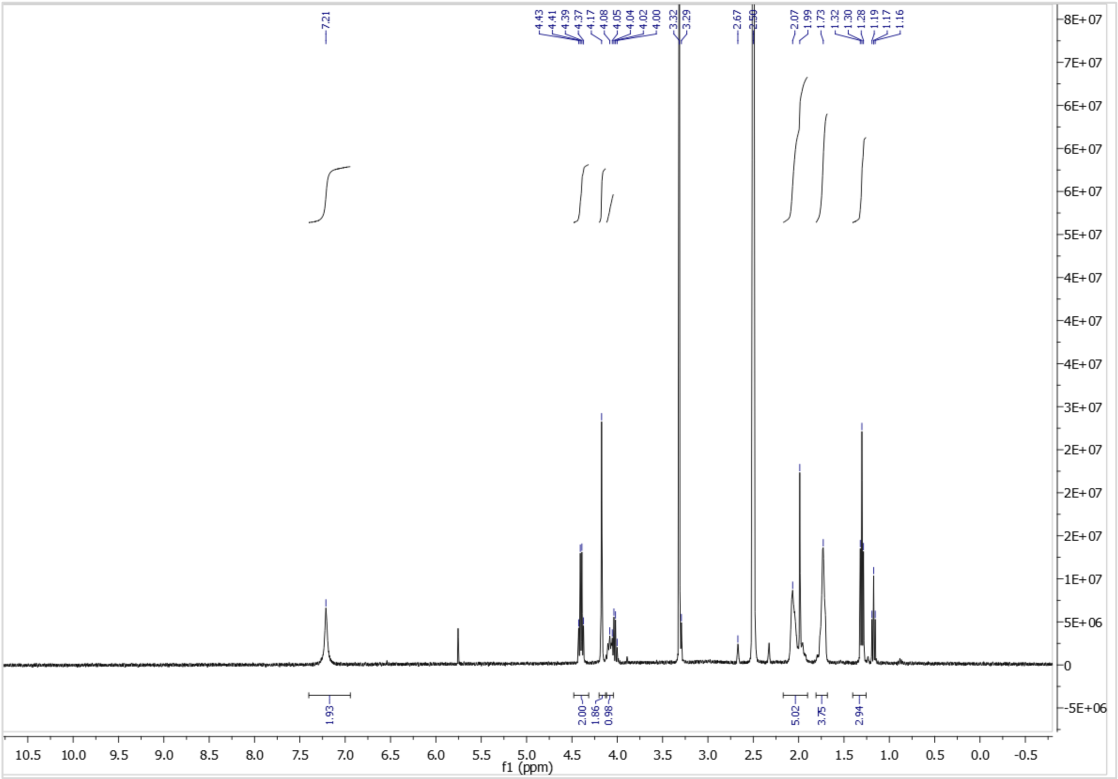

33

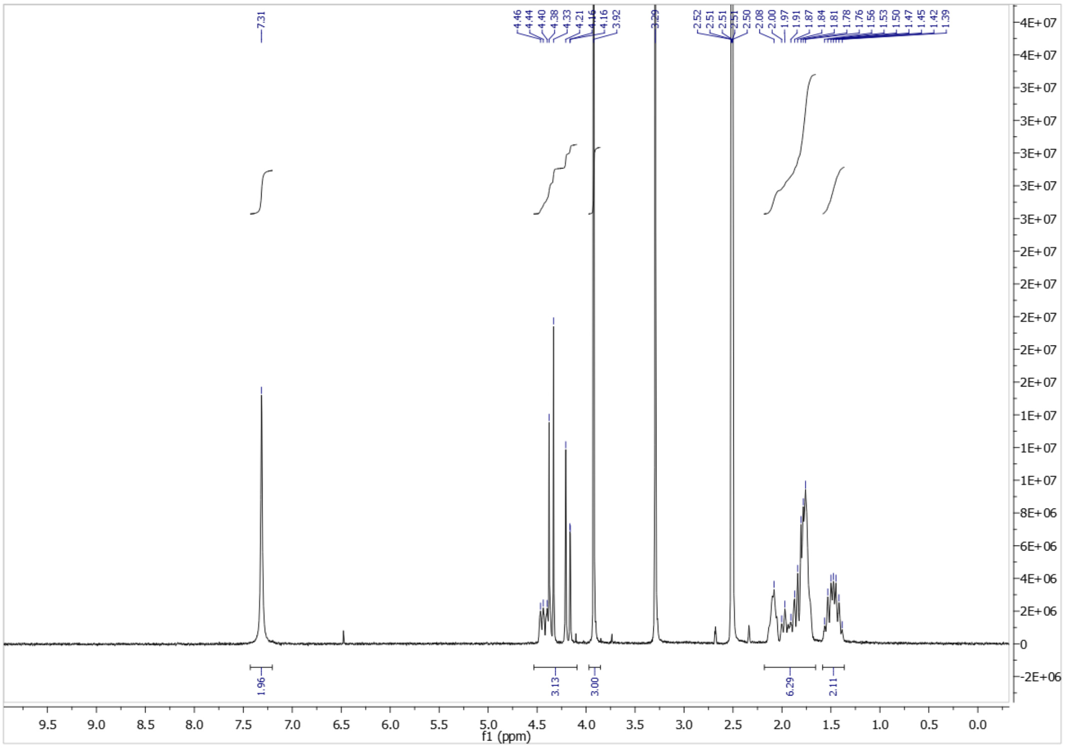
7.31
4.46 4.44 4.40 4.38 4.33 4.21 4.16 4.16 3.92
3.29
2.52 2.51 2.51 2.51 2.50 2.08 2.00 1.97 1.91 1.87 1.84 1.81 1.78 1.76 1.56 1.53 1.50 1.47 1.45 1.42 1.39
1.96
3.13
3.00
6.29
2.11
f1 (ppm)

34

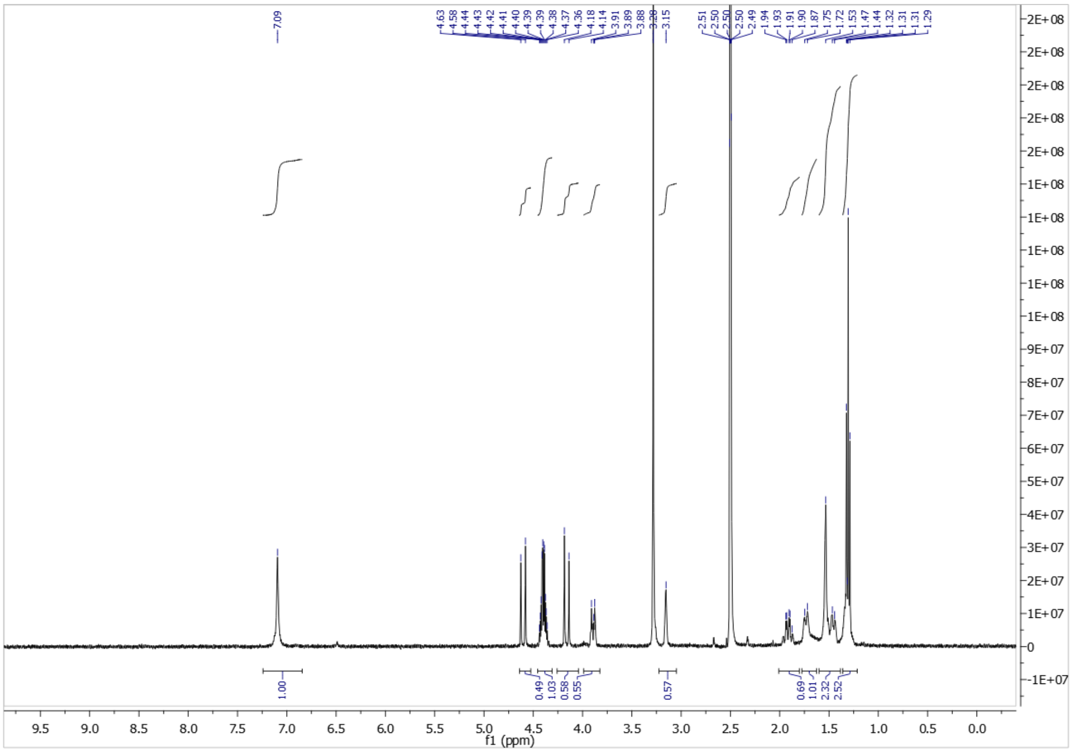
7.09
4.63 4.58 4.44 4.43 4.42 4.41 4.40 4.39 4.38 4.37 4.36 4.18 4.14 3.91 3.89 3.88
3.28
3.15
2.51 2.50 2.50 2.50 2.49 1.94 1.93 1.91 1.90 1.87 1.75 1.72 1.53 1.47 1.44 1.32 1.31 1.29
1.00
0.49
1.03
0.58
0.55
0.57
0.69
1.01
2.32
2.52
f1 (ppm)

35

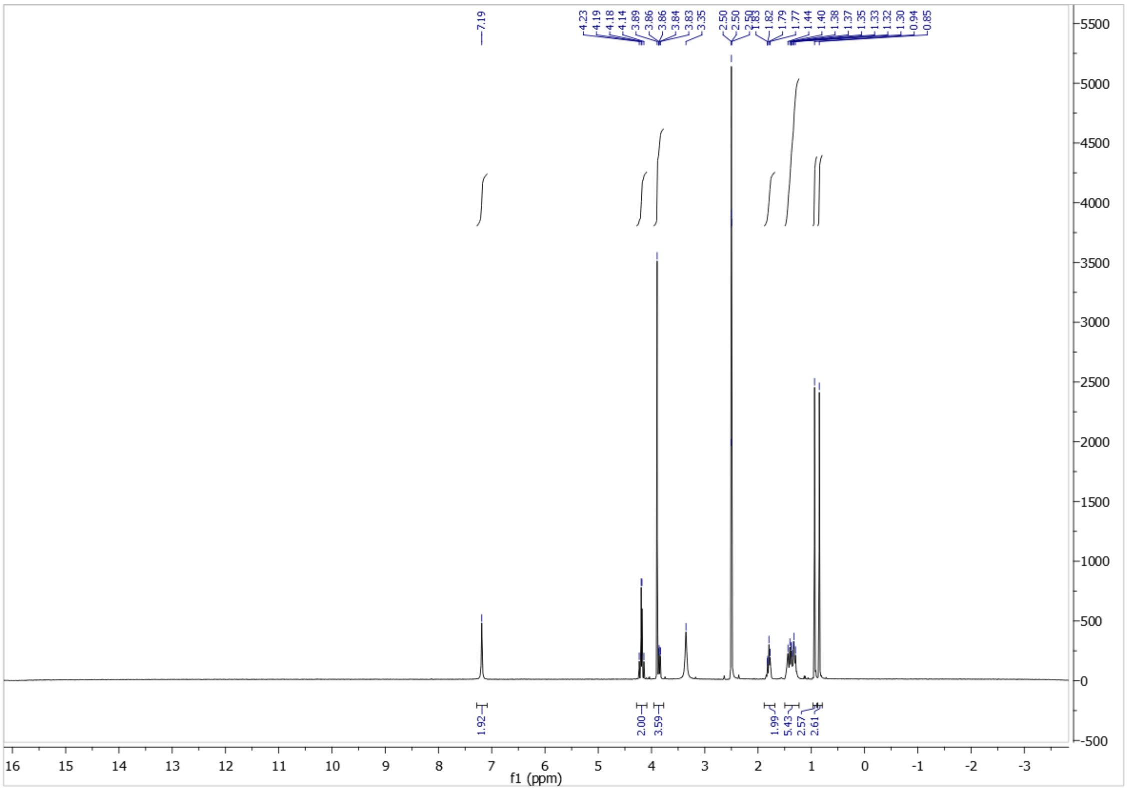

38

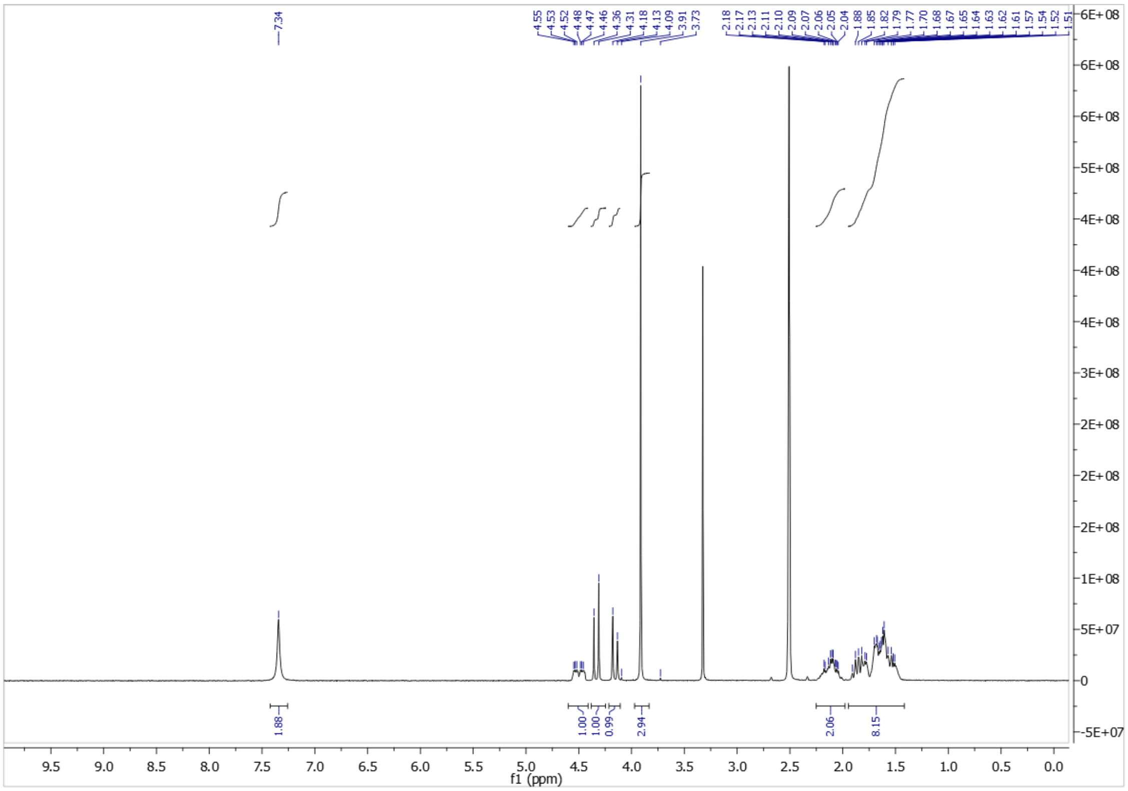

39

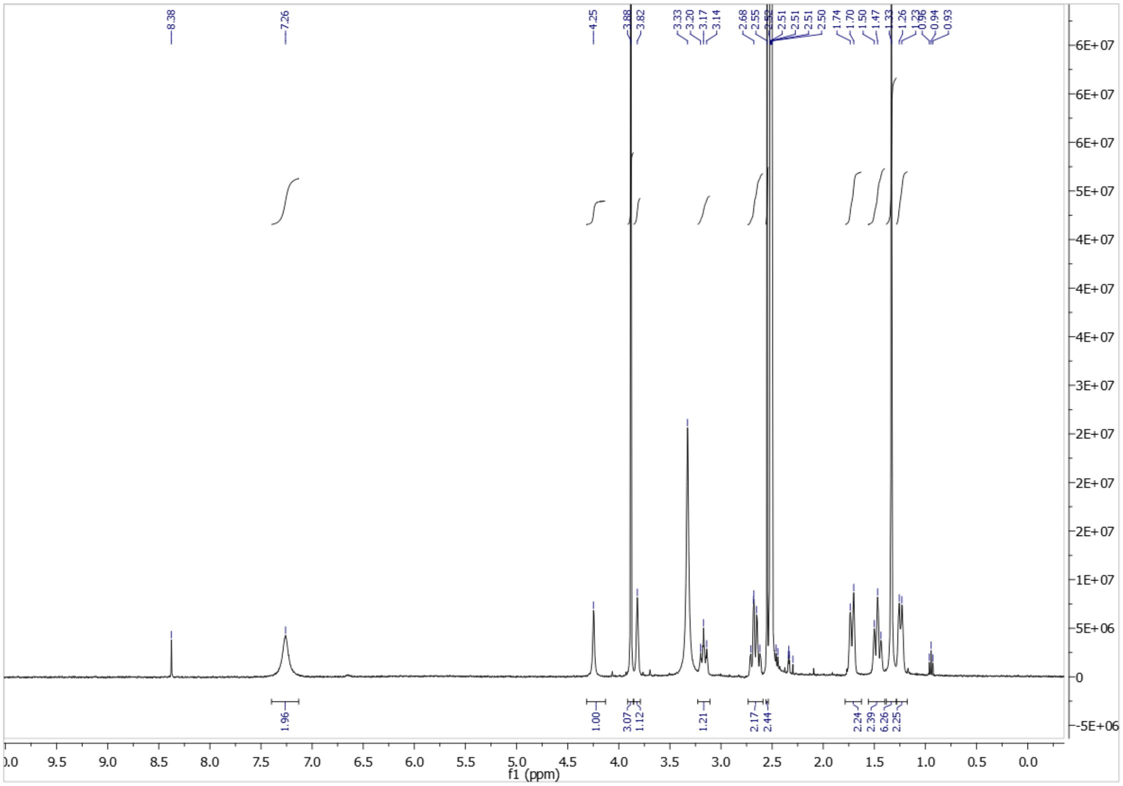

40

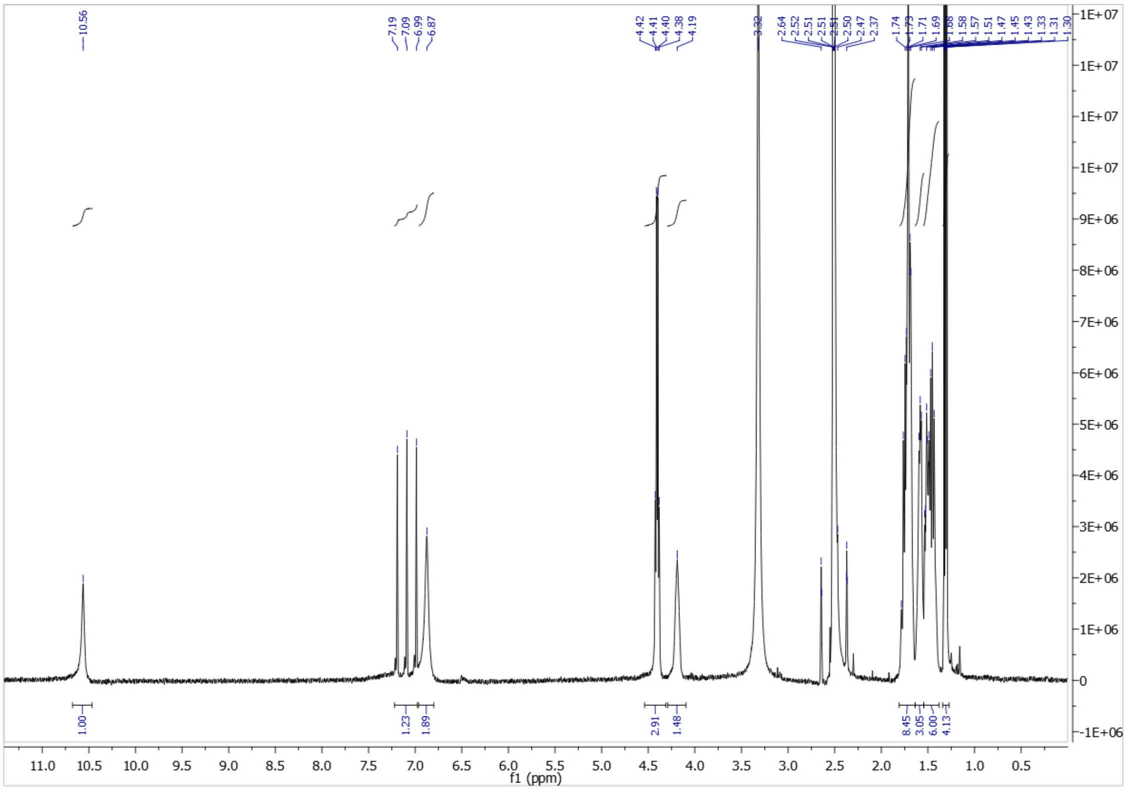

41

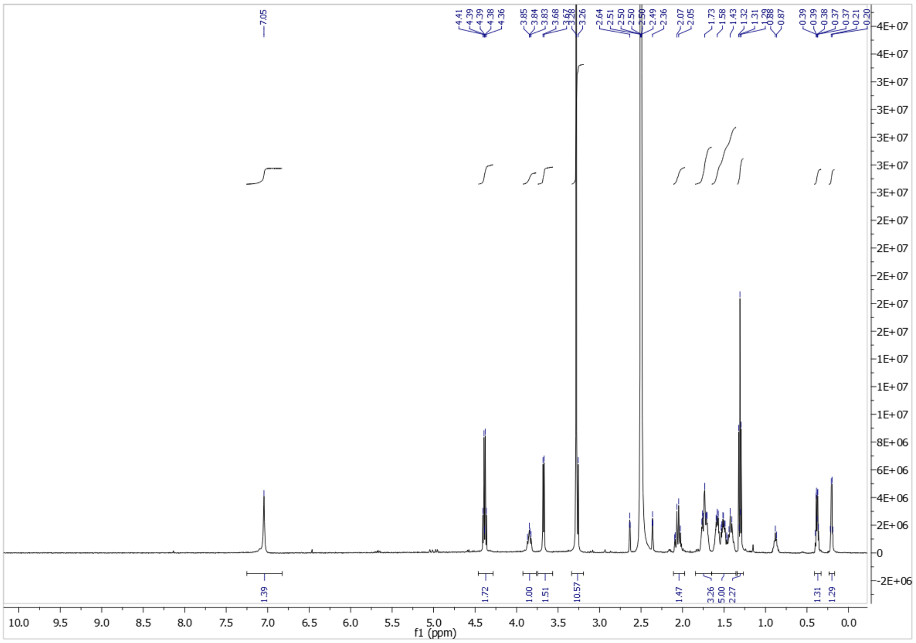

Integration did not correlate as expected. HRMS included to confirm purity level was acceptable for initial profiling.

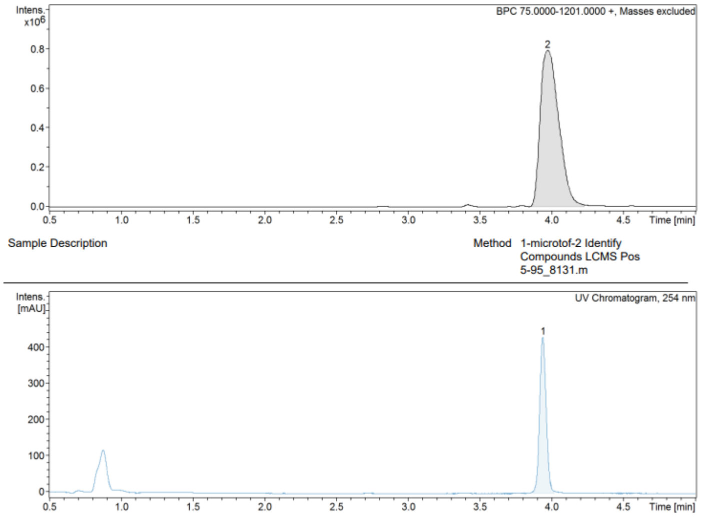

RT [min]	Area Frac. %	Chromatogram
3.9	100.00	UV Chromatogram, 254 nm
4.0	100.00	BPC 75.0000-1201.0000 +, Masses excluded

Cmpd 2, 4.0 min

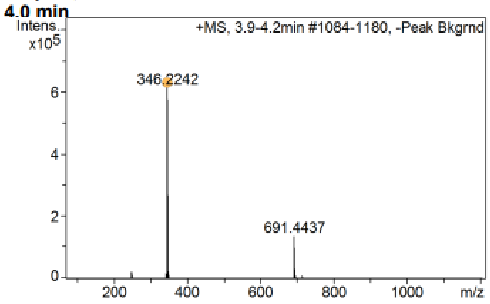

42

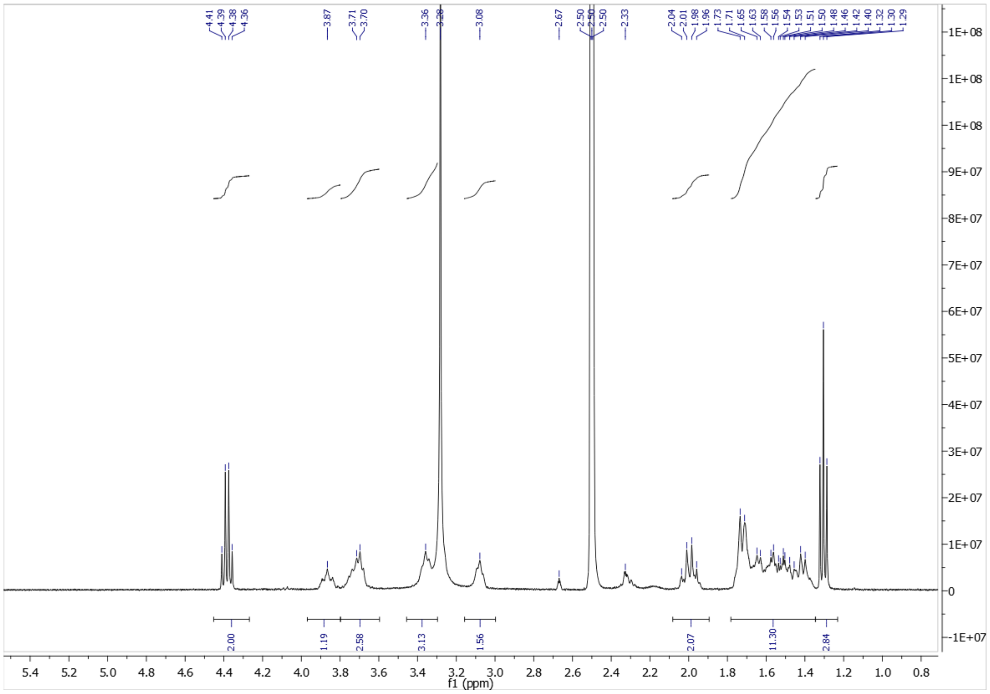

Integration did not correlate as expected. HRMS included to confirm purity level was acceptable for initial profiling.

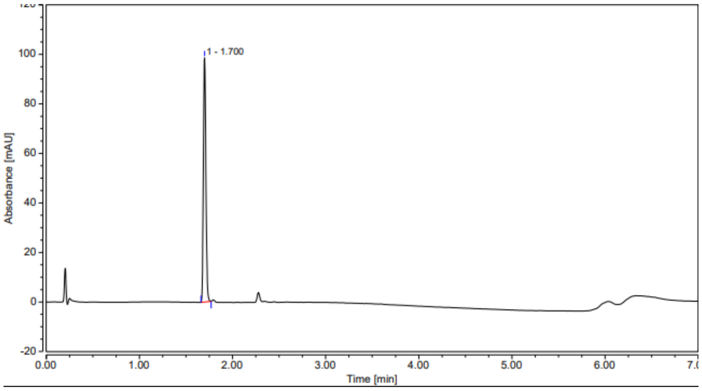

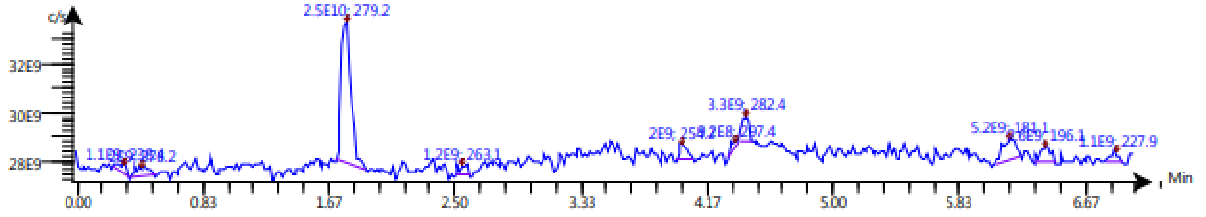

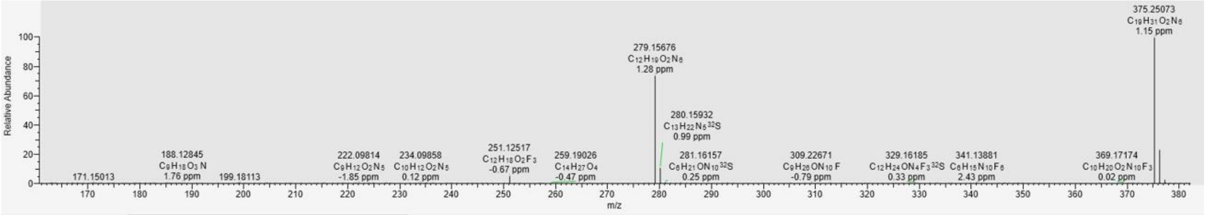

43

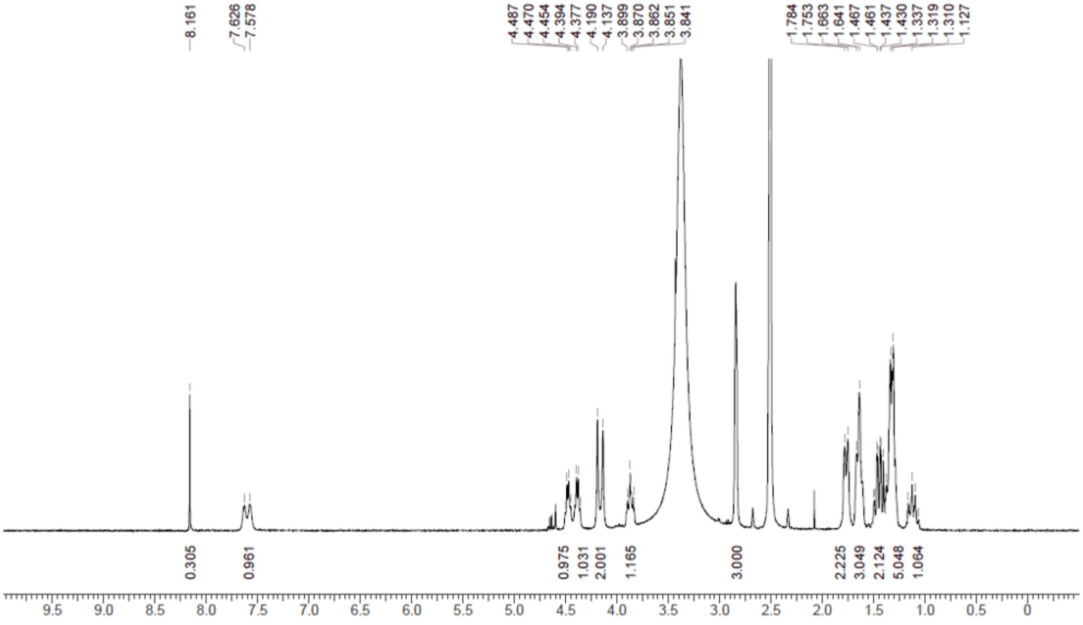
8.161
7.626
7.578
4.487
4.470
4.454
4.394
4.377
4.190
4.137
3.899
3.870
3.862
3.851
3.841
1.784
1.753
1.663
1.641
1.467
1.461
1.437
1.430
1.337
1.319
1.310
1.127
0.305
0.961
0.975
1.031
2.001
1.165
3.000
2.225
3.049
2.124
5.048
1.064
9.5 9.0 8.5 8.0 7.5 7.0 6.5 6.0 5.5 5.0 4.5 4.0 3.5 3.0 2.5 2.0 1.5 1.0 0.5 0

44

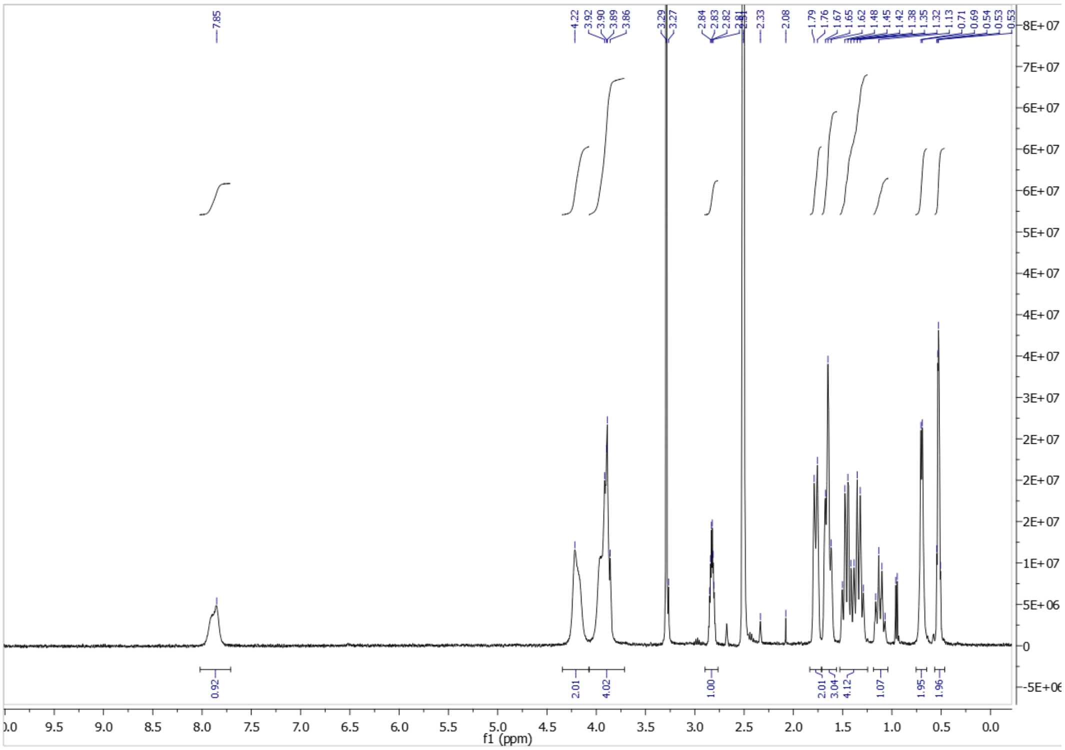
7.85
4.22
3.92
3.90
3.89
3.86
3.29
3.27
2.84
2.83
2.82
2.81
2.33
2.08
1.79
1.76
1.67
1.65
1.62
1.48
1.45
1.42
1.38
1.35
1.32
1.13
0.71
0.69
0.54
0.53
0.53
0.92
2.01
4.02
1.00
2.01
3.04
4.12
1.07
1.95
1.96
f1 (ppm)

45

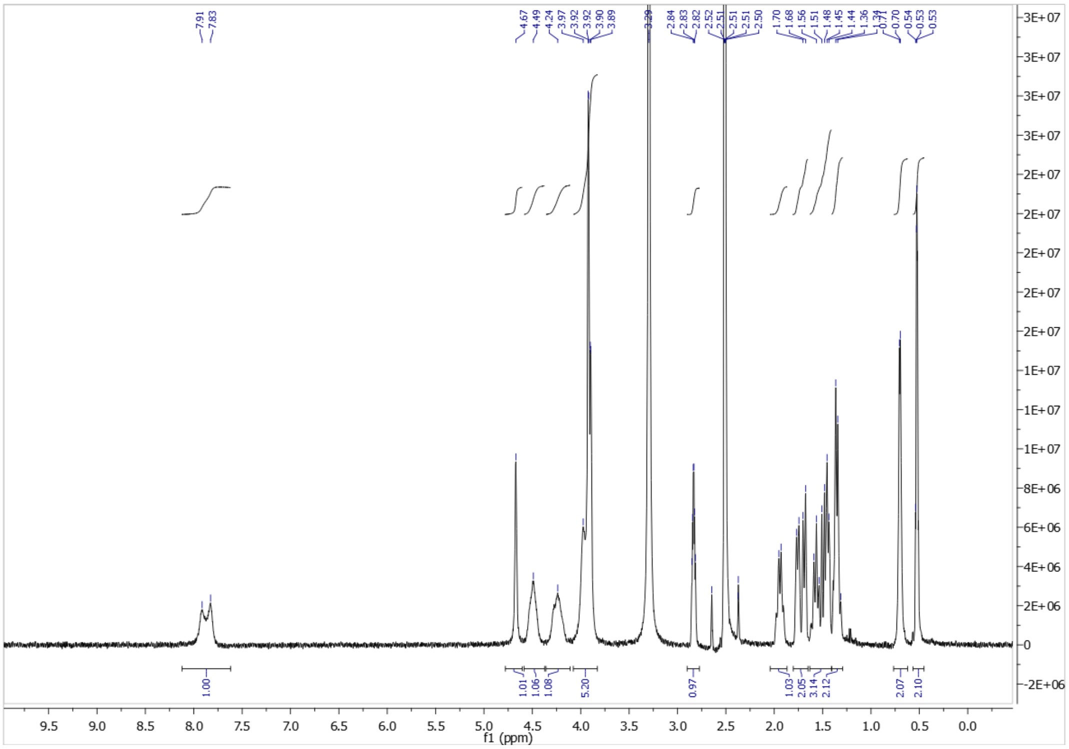

48

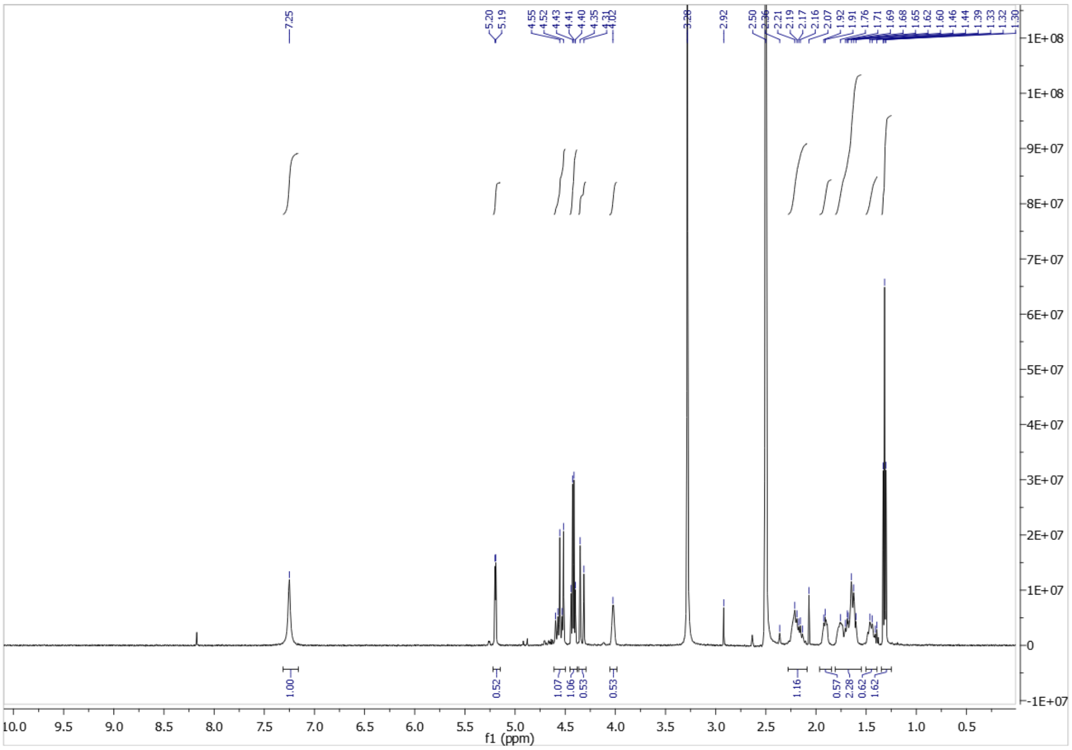

13C NMR spectra for compounds progressed to in vivo studies

8

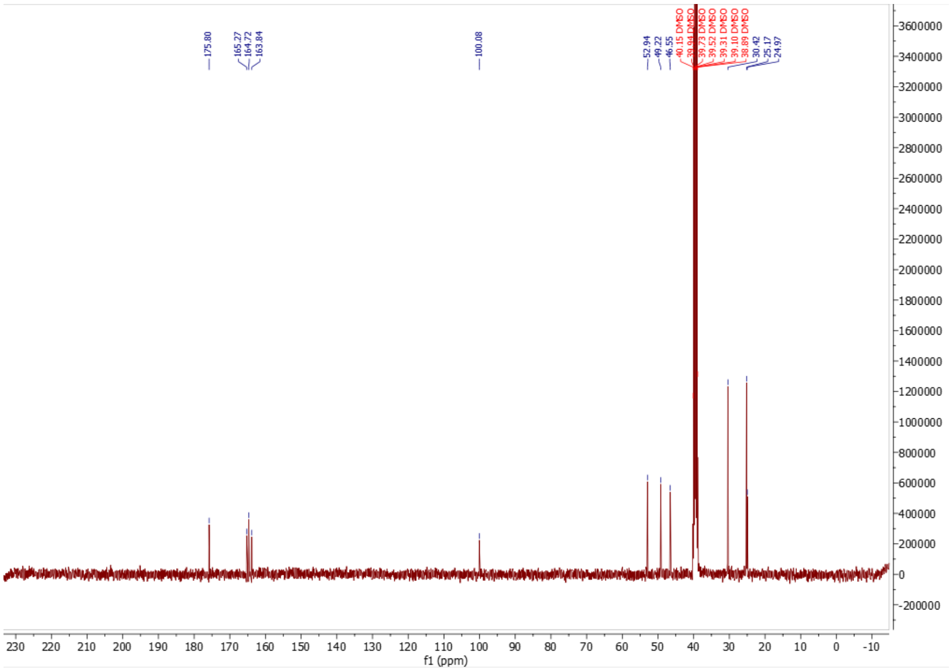

11

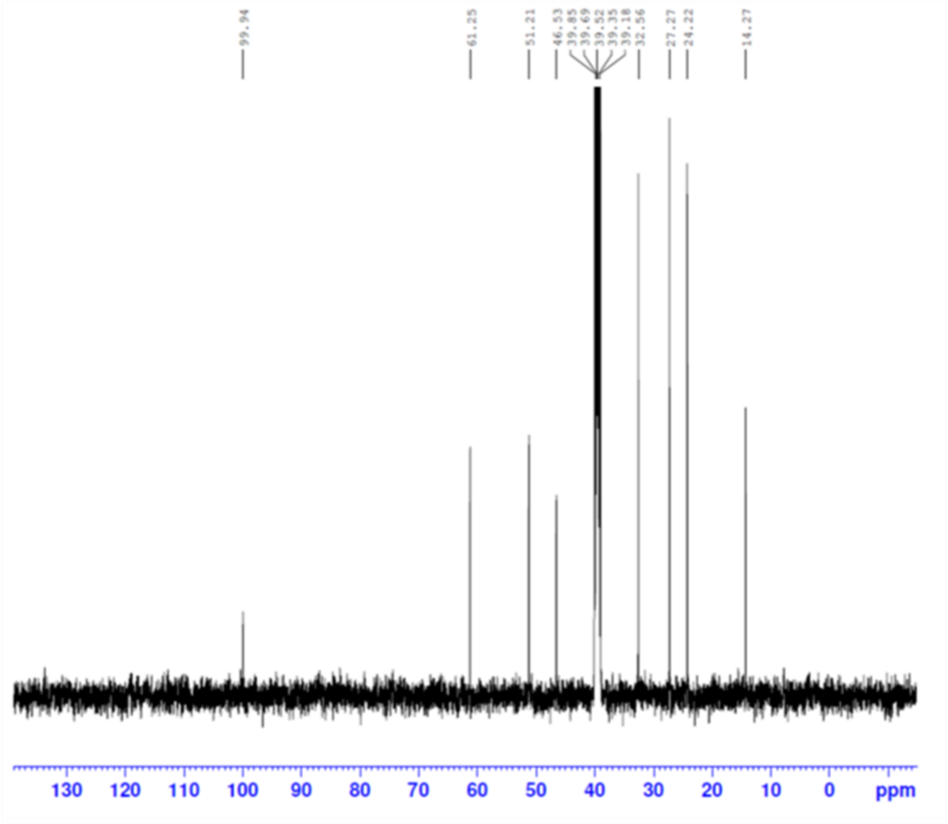

25

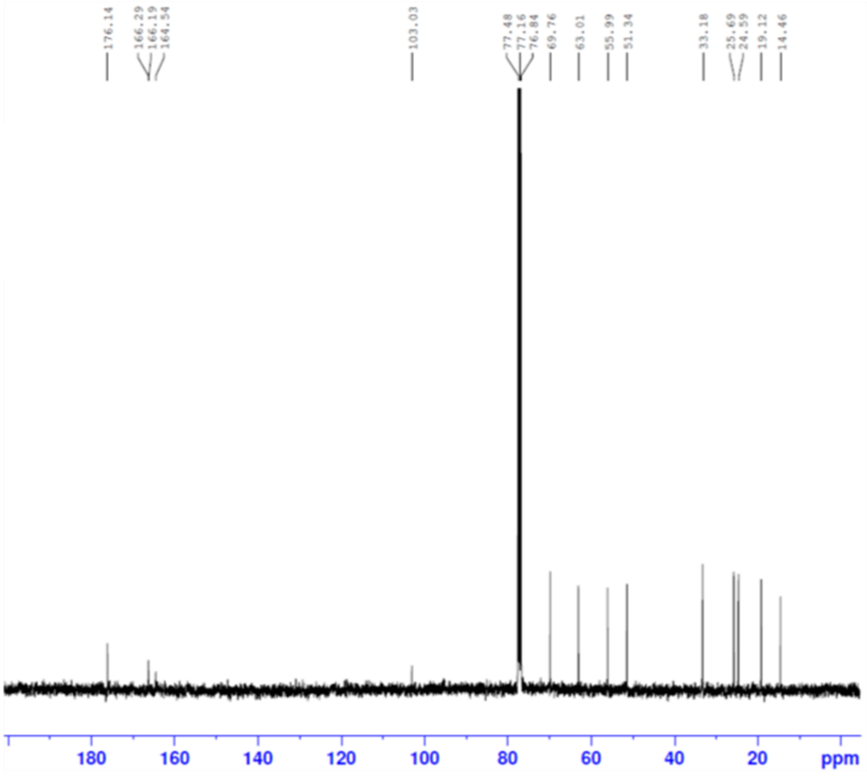
176.14
166.29
166.19
164.54
103.03
77.48
77.16
76.84
69.76
63.01
55.99
51.34
33.18
25.69
24.59
19.12
14.46
180
160
140
120
100
80
60
40
20
ppm

32

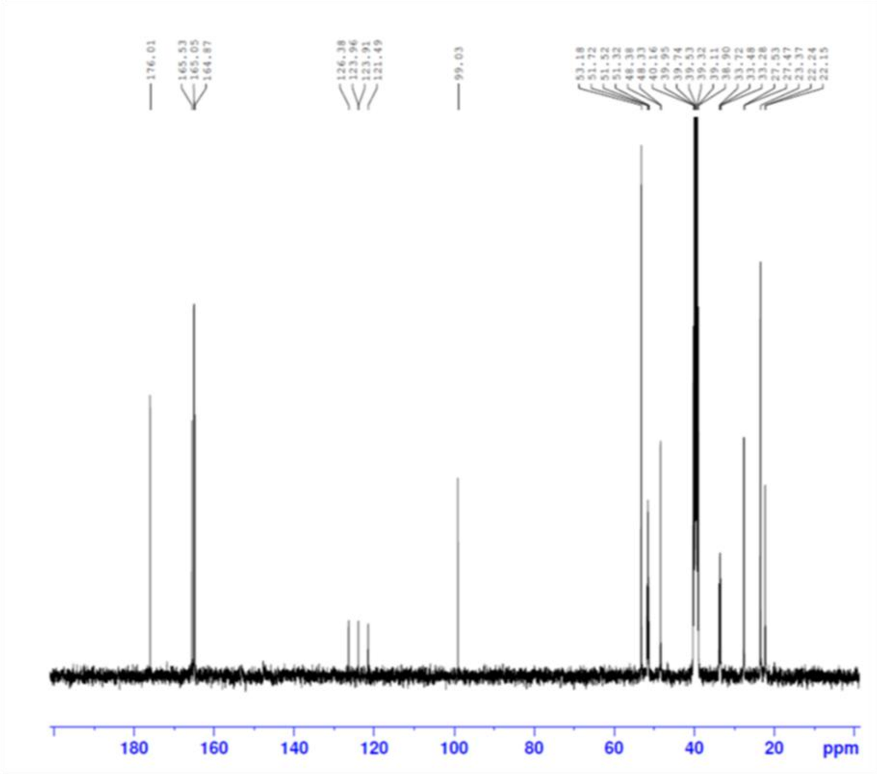
176.01
165.53
165.05
164.87
126.38
123.96
123.91
121.49
99.03
180
160
140
120
100
80
60
40
20
ppm

37

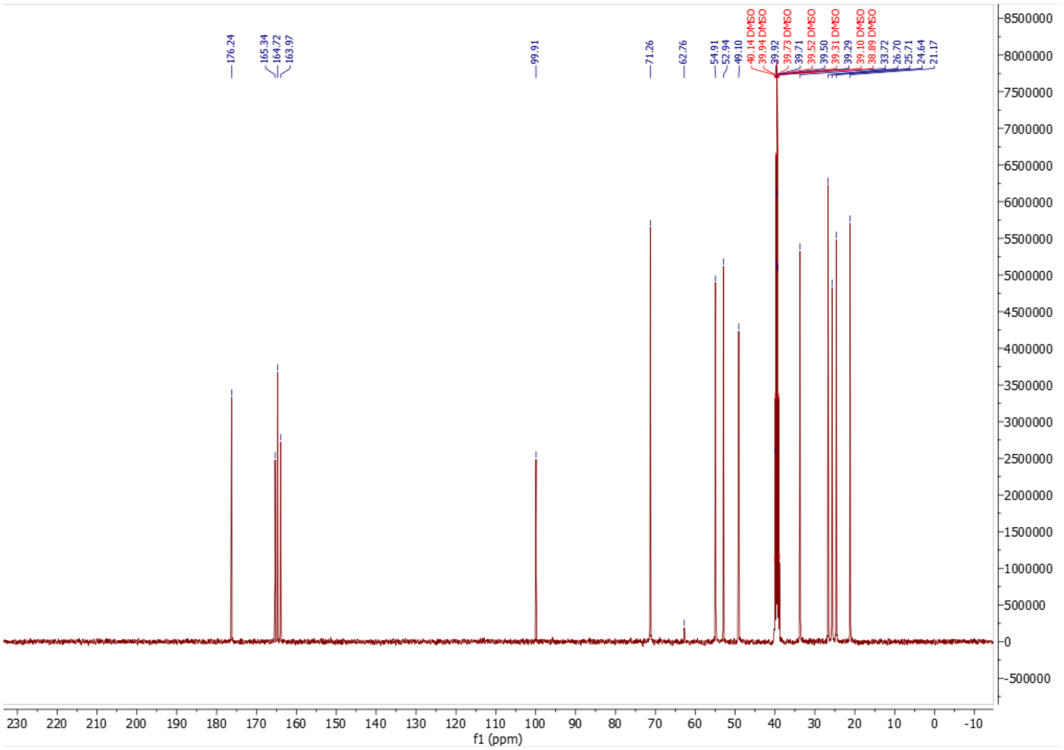
176.24
165.34
164.72
163.97
99.91
71.26
62.76
54.91
52.94
49.10
40.14 DMSO
39.94 DMSO
39.92
39.73 DMSO
39.71
39.52 DMSO
39.50
39.31 DMSO
39.29
39.10 DMSO
38.89 DMSO
33.72
26.70
25.71
24.64
21.17
f1 (ppm)

38

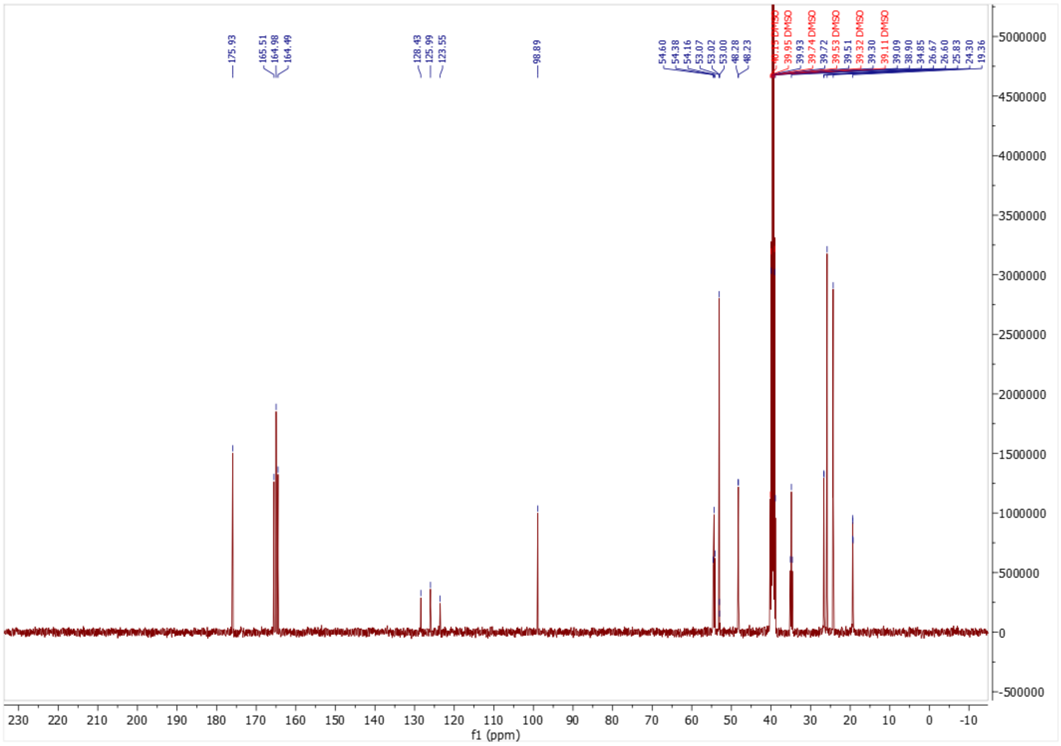
175.93
165.51
164.98
164.49
128.43
125.99
123.55
98.89
54.60
54.38
54.16
53.07
53.02
53.00
48.28
48.23
39.95 DMSO
39.93
39.74 DMSO
39.72
39.53 DMSO
39.51
39.32 DMSO
39.30
39.11 DMSO
39.09
38.90
34.85
26.67
26.60
25.83
24.30
19.36
f1 (ppm)

46

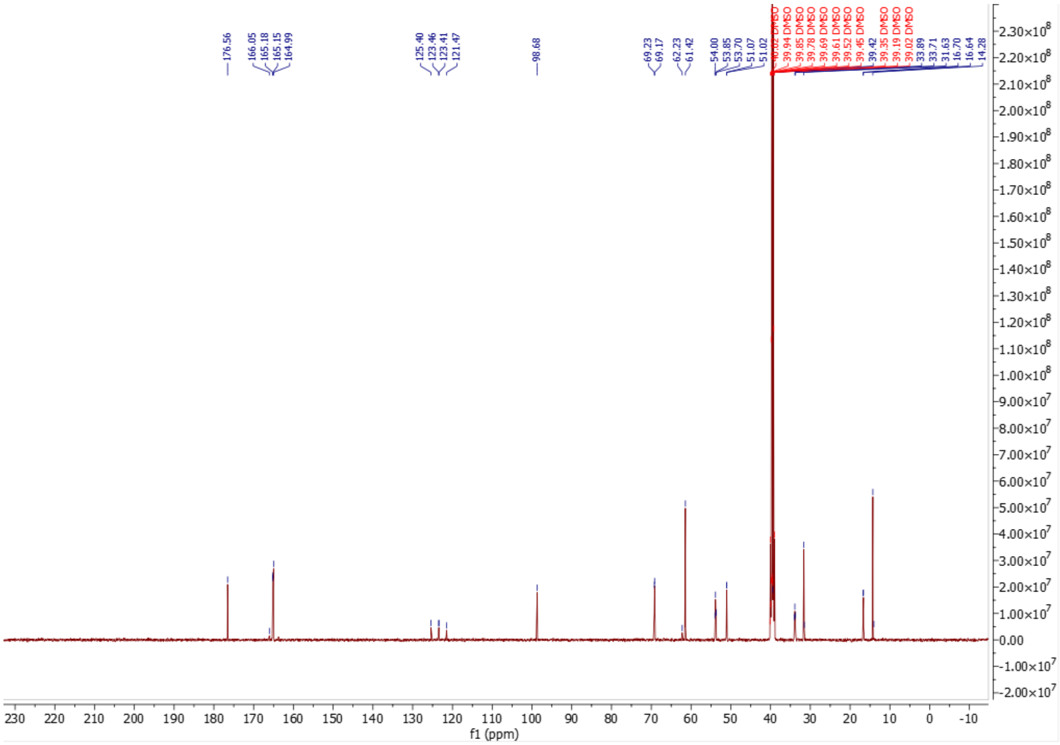

47

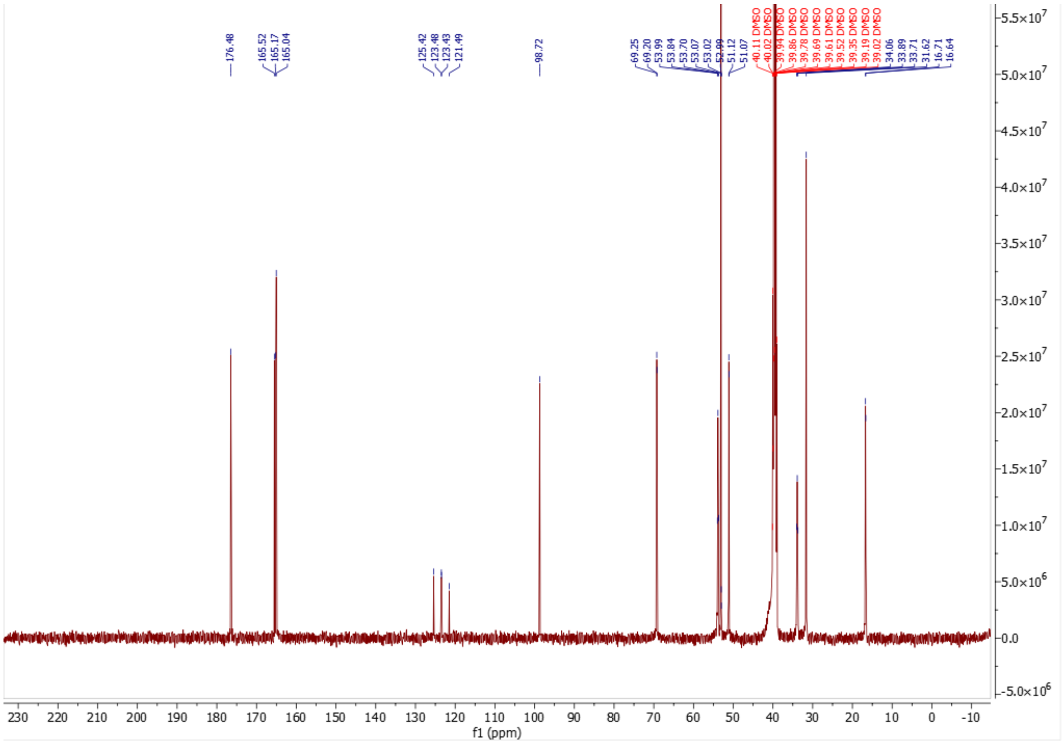

48

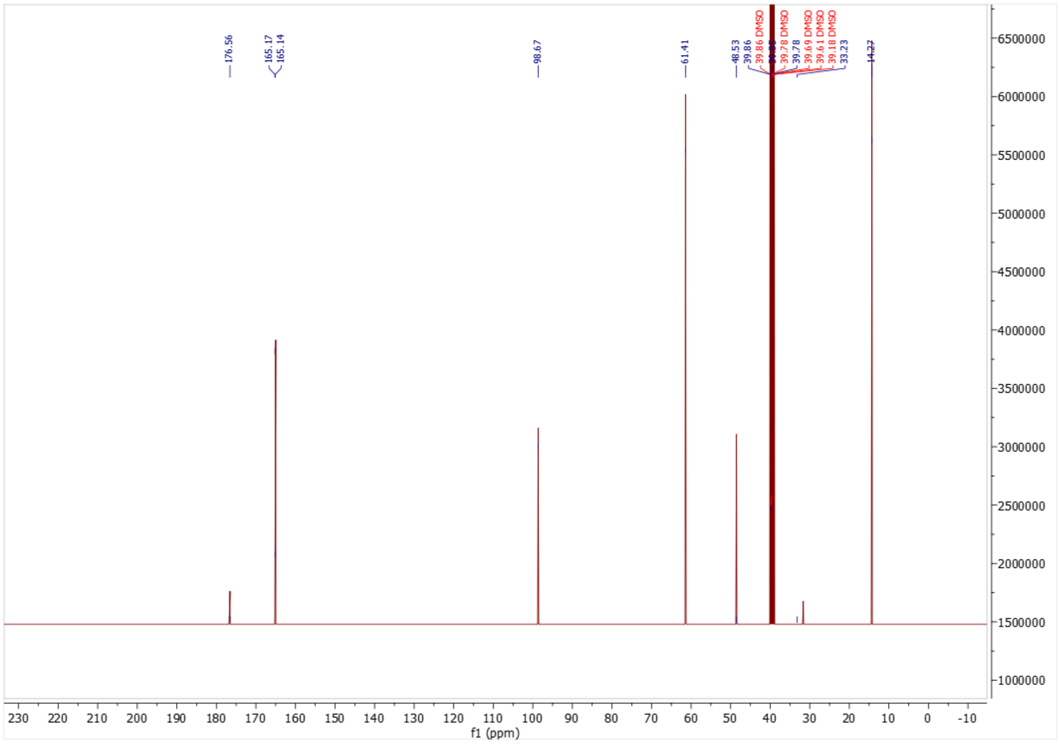
176.56
165.17
165.14
98.67
61.41
48.53
39.86
39.78
33.23
14.27
f1 (ppm)

49

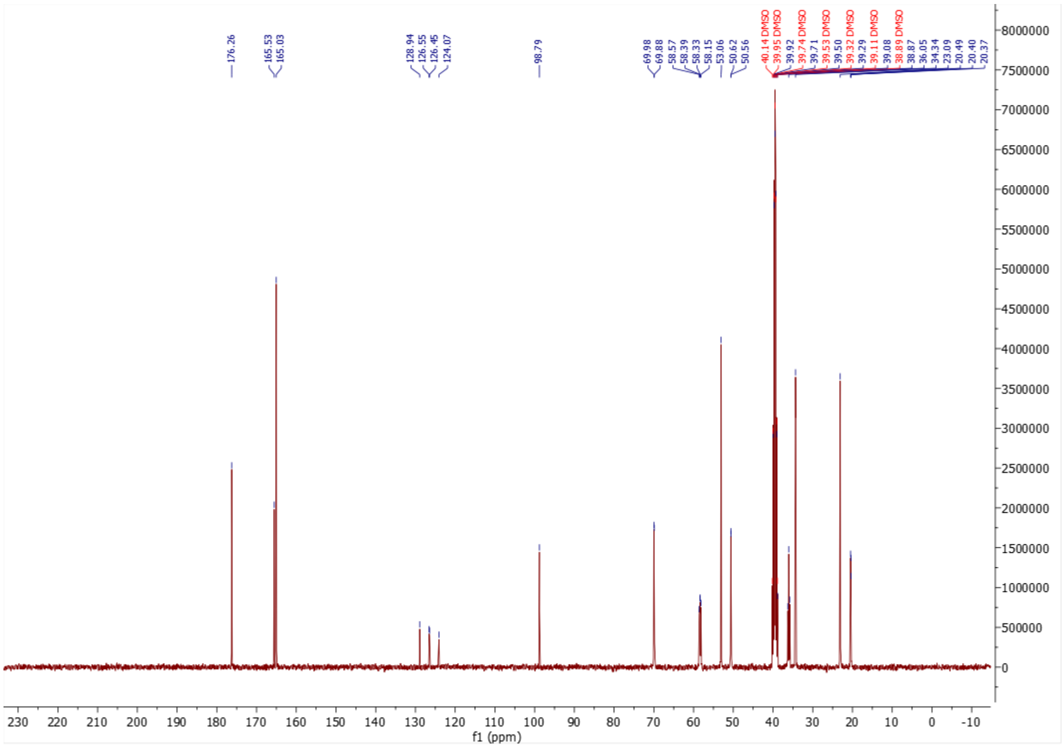
176.26
165.53
165.03
128.94
126.55
126.45
124.07
98.79
69.98
69.88
58.57
58.39
58.33
58.15
53.06
50.62
50.56
f1 (ppm)

HRMS Spectra for compounds progressed to in vivo studies

8

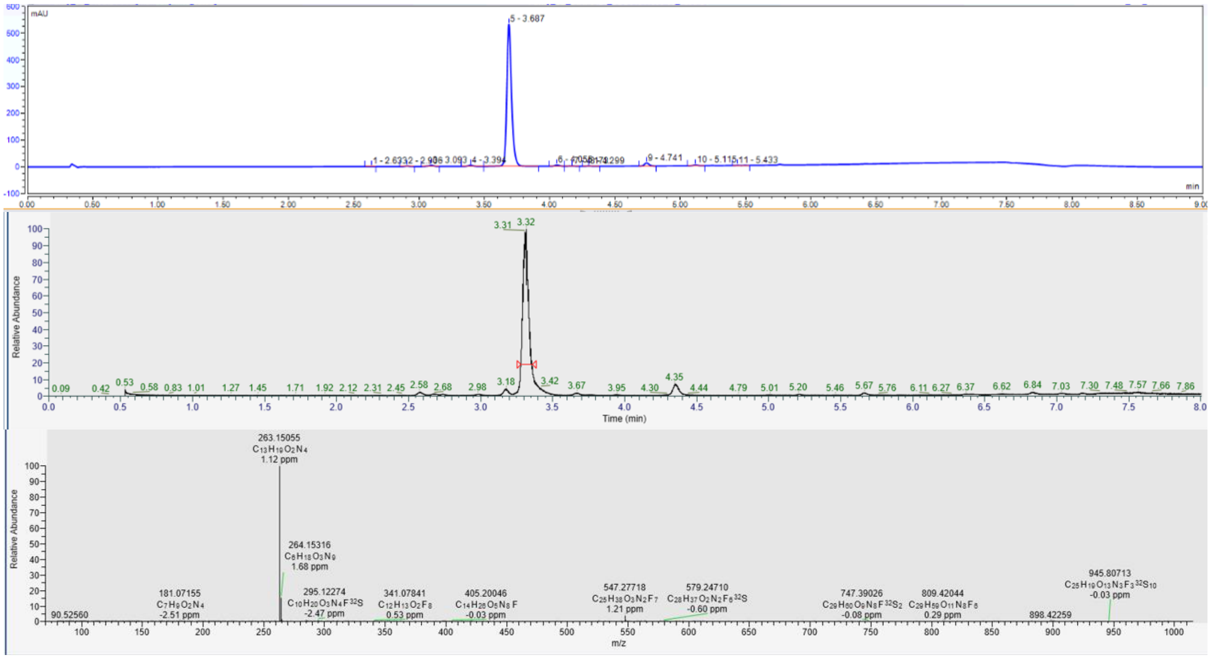

10

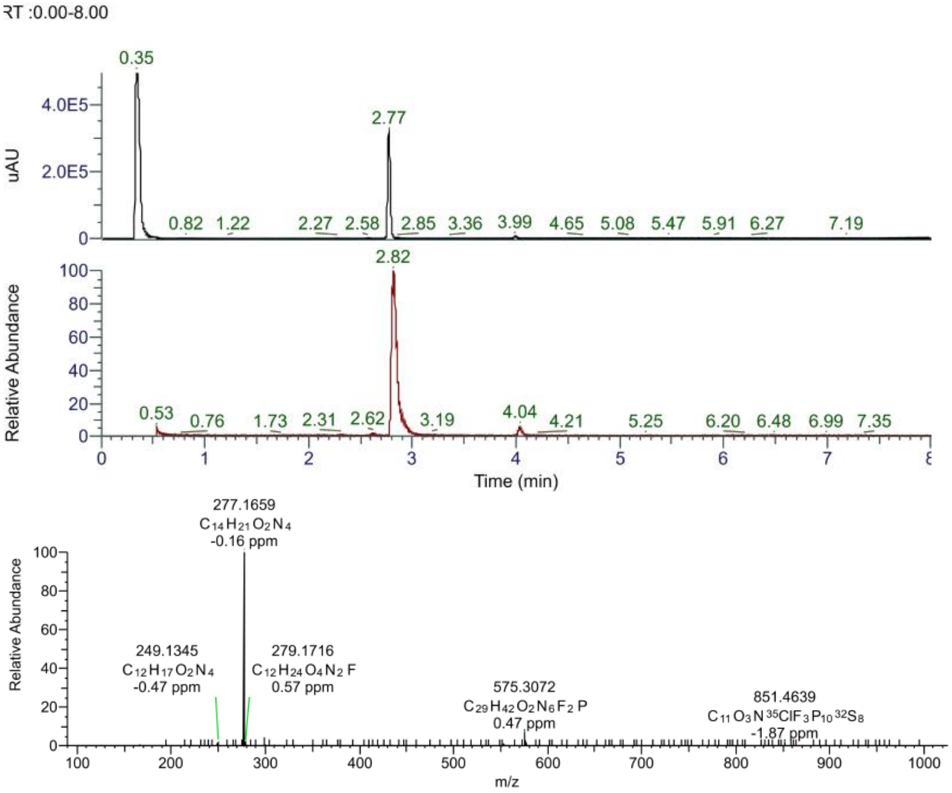

11

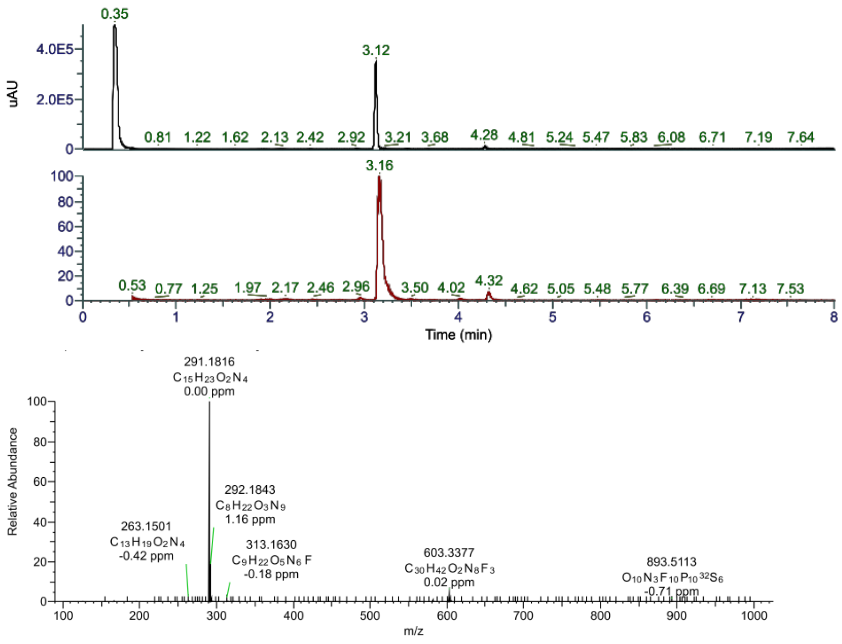
0.35
4.0E5
uAU
2.0E5
3.12
0.81 1.22 1.62 2.13 2.42 2.92 3.21 3.68 4.28 4.81 5.24 5.47 5.83 6.08 6.71 7.19 7.64
3.16
0.53 0.77 1.25 1.97 2.17 2.46 2.96 3.50 4.02 4.32 4.62 5.05 5.48 5.77 6.39 6.69 7.13 7.53
Time (min)
291.1816
C15H23O2N4
0.00 ppm
Relative Abundance
292.1843
C8H22O3N9
1.16 ppm
263.1501
C13H19O2N4
-0.42 ppm
313.1630
C9H22O5N6F
-0.18 ppm
603.3377
C30H42O2N8F3
0.02 ppm
893.5113
O10N3F10P10 32S6
-0.71 ppm
m/z

25

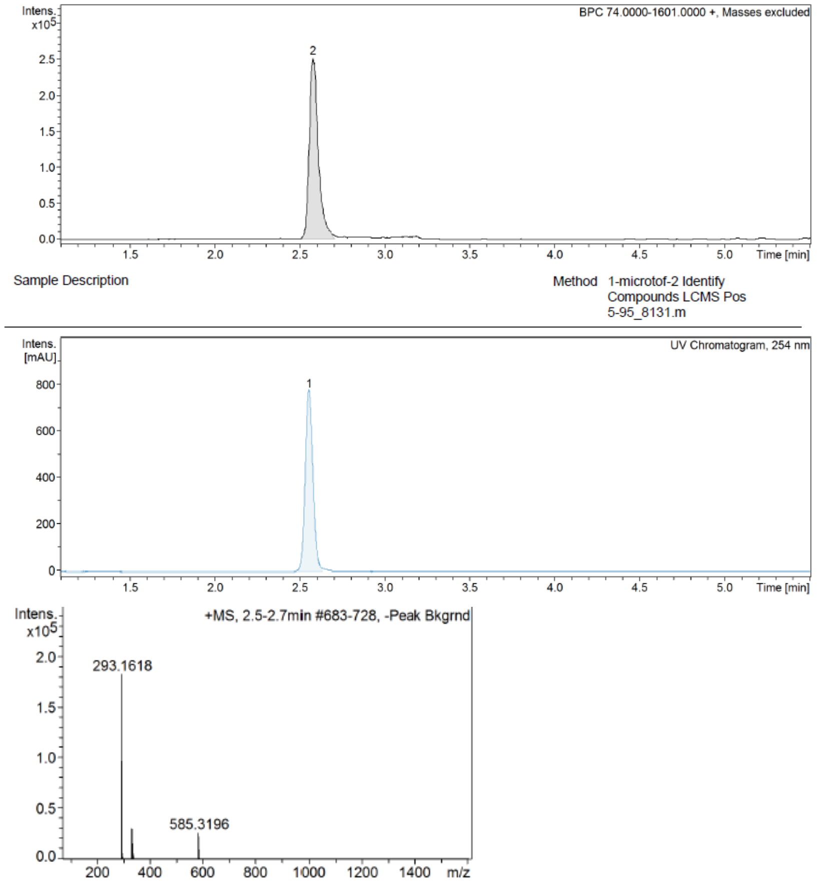
Intens.
x10^5
BPC 74.0000-1601.0000 +, Masses excluded
2
Time [min]
Sample Description
Method 1-microtof-2 Identify
Compounds LCMS Pos
5-95_8131.m
Intens.
[mAU]
UV Chromatogram, 254 nm
1
Time [min]
Intens.
x10^5
+MS, 2.5-2.7min #683-728, -Peak Bkgrnd
293.1618
585.3196
m/z

32

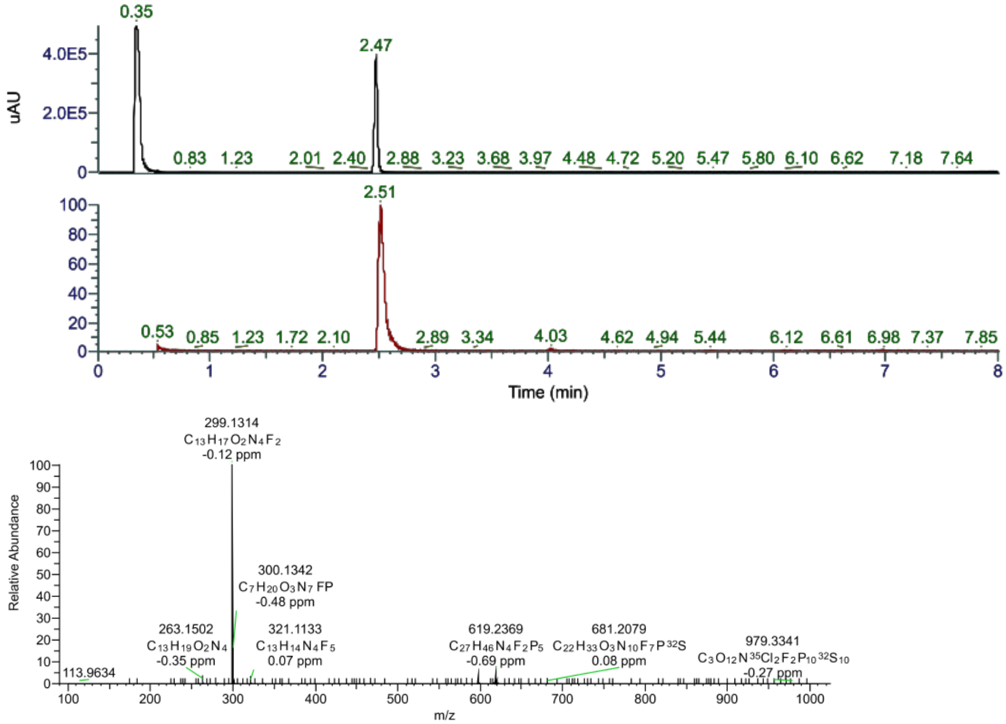
0.35
2.47
4.0E5
2.0E5
uAU
0.83 1.23 2.01 2.40 2.88 3.23 3.68 3.97 4.48 4.72 5.20 5.47 5.80 6.10 6.62 7.18 7.64
2.51
0.53 0.85 1.23 1.72 2.10 2.89 3.34 4.03 4.62 4.94 5.44 6.12 6.61 6.98 7.37 7.85
Time (min)
299.1314
C13H17O2N4F2
-0.12 ppm
300.1342
C7H20O3N7FP
-0.48 ppm
263.1502
C13H19O2N4
-0.35 ppm
321.1133
C13H14N4F5
0.07 ppm
619.2369
C27H46N4F2P5
-0.69 ppm
681.2079
C22H33O3N10F7P32S
0.08 ppm
979.3341
C3O12N35Cl2F2P10 32S10
-0.27 ppm
113.9634
Relative Abundance
m/z

37

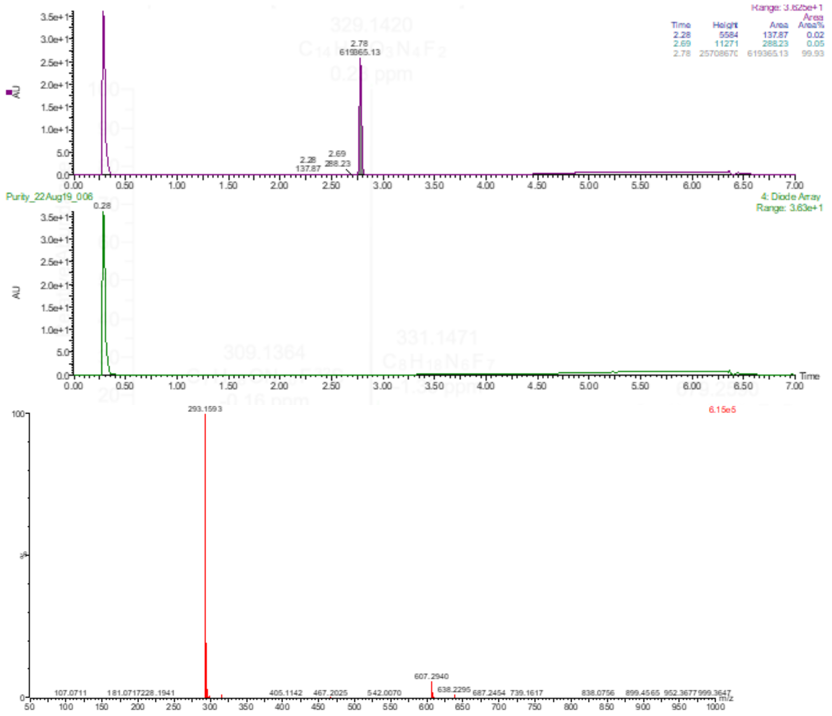
2.78
2.28
2.69
AU
Purity_22Aug19_006
4: Diode Array
0.28
Time
293.1593
6.15e5
607.2940

38

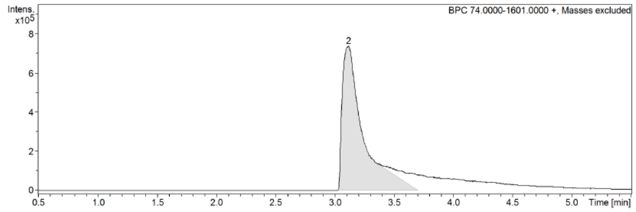

Sample Description

Method 1-microtof-2 Identify Compounds LCMS Pos 5-95_8131.m

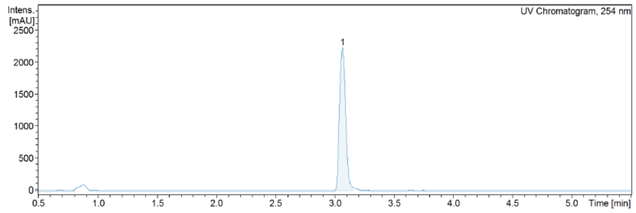

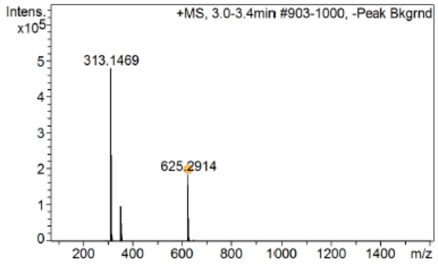

46

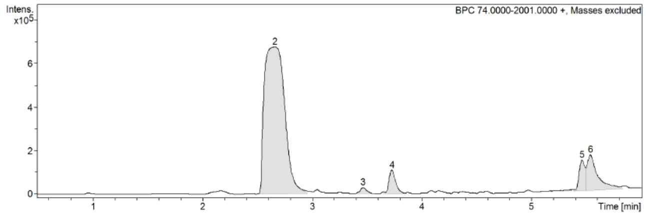

Sample Description Lyo and dried

Method 3-microtof-2 Identify Compounds BASIC LCMS Pos 5-95_8132.m

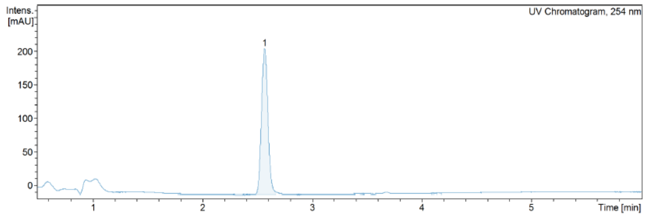

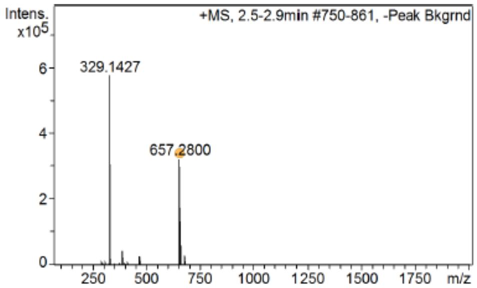

47

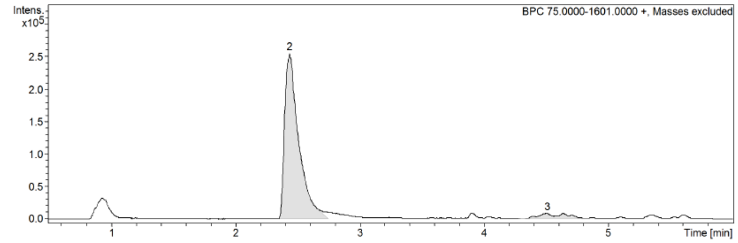

Sample Description

Method 1-microtof-2 Identify Compounds LCMS Pos 5-95_8131.m

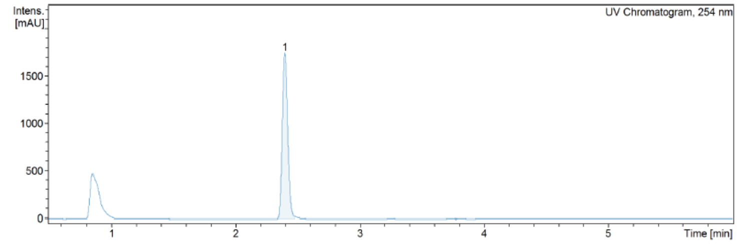

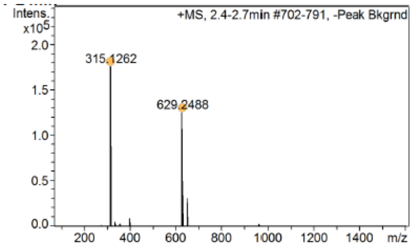

48

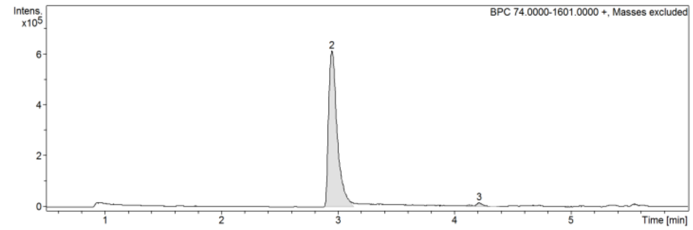

Sample Description

Method 1-microtof-2 Identify Compounds LCMS Pos 5-95_8131.m

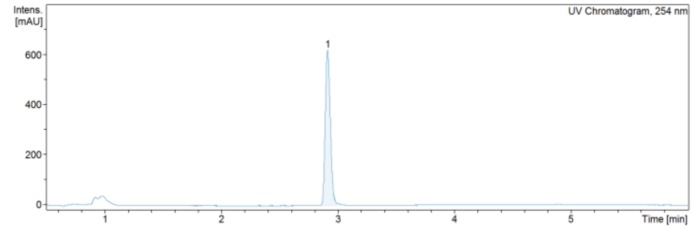

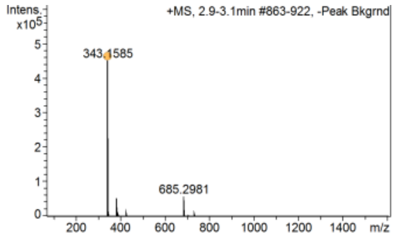

49

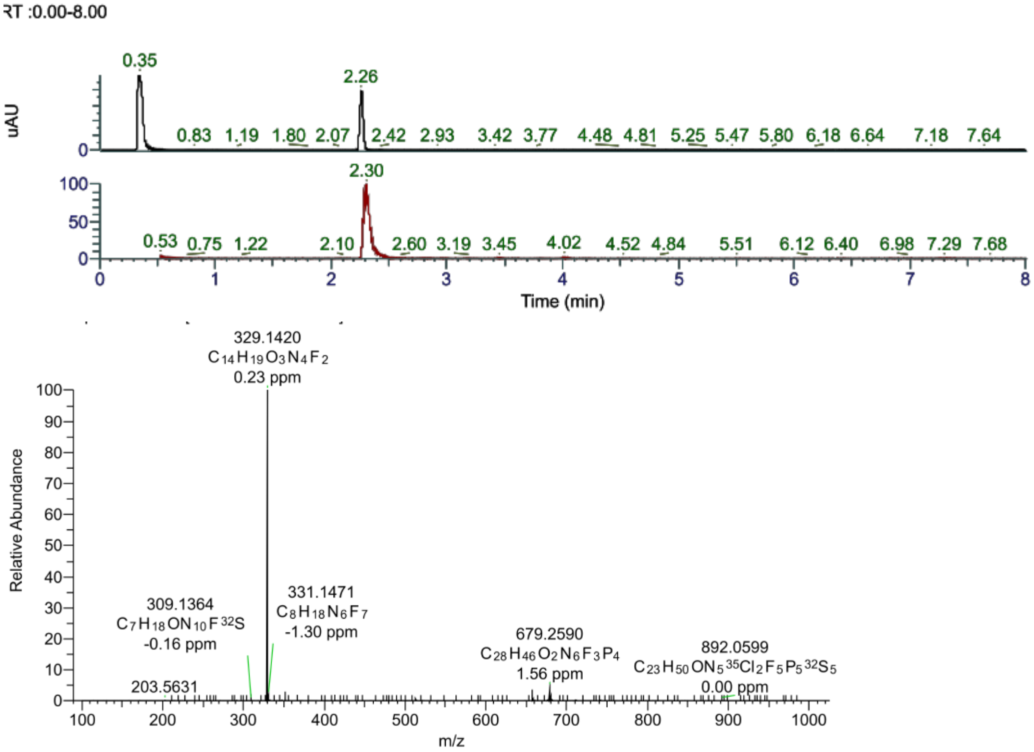

RT :0.00-8.00
uAU
0.35
2.26
0.83 1.19 1.80 2.07 2.42 2.93 3.42 3.77 4.48 4.81 5.25 5.47 5.80 6.18 6.64 7.18 7.64
2.30
0.53 0.75 1.22 2.10 2.60 3.19 3.45 4.02 4.52 4.84 5.51 6.12 6.40 6.98 7.29 7.68
Time (min)
329.1420
C14H19O3N4F2
0.23 ppm
Relative Abundance
309.1364
C7H18ON10F32S
-0.16 ppm
331.1471
C8H18N6F7
-1.30 ppm
679.2590
C28H46O2N6F3P4
1.56 ppm
892.0599
C23H50ON535Cl2F5P532S5
0.00 ppm
203.5631
m/z

Supplementary Methods

In vivo metabolite identification of 11.

Sample Preparation: Blood samples (30 µL) from the 10 mg/kg orally dosing PK study were vortexed with LCMS grade acetonitrile (90 µL) and centrifuged (13k rpm for 5 min) to remove any precipitated proteins. Supernatant (80 µL) from each sample was then added to 80 µL water (Milli-Q) and vortexed in preparation for LCMS analysis.

Analysis: Samples were analysed using a Waters Acquity UPLC with a diode array detector and Waters Xevo Q-TOF mass spectrometer. Reversed phase separation of the metabolites was accomplished using gradient elution method. The following elution profile was used: Eluent A, water plus 0.01% formic acid. Eluent B, acetonitrile plus 0.01% formic acid. Hold at 5% A for 0.5 min. Linearly increase to 40% B over 3.5 min. Then linearly increase to 95% B over another 2 min. One minute re-equilibration. Total run time was 7 min. Flow rate was 0.5 mL/min. Separation was achieved using a Waters BEH C18 column (50 x 2.1 mm, 1.7 µm particle size. 130 Å pore size, Cat. No. 186002350). The column temperature was 40°C. The UV response was monitored from 200-400 nm but not used in this study.

The mass spectrometer was operated using an electrospray (ESi) source in positive mode only. The source temperature was set to 120°C and the capillary voltage to 1.5 kV. The sample cone had an applied potential of 40V. For initial metabolite identification MS^2 (ms/ms) spectra were obtained using the MS^e mode on the Waters QToF spectrometer. Possible metabolites were then confirmed by re-injecting the test sample and collecting targeted MS^2 spectra, using the retention time and mass of the metabolite. A ramped collision energy (20, 30 and 40 V) was used for fragmentation of the metabolites by CID. These spectra were used for metabolite identification.
